# When less is more: cell size reduction as a metabolic lifeline in *Mycoplasma gallisepticum*

**DOI:** 10.3389/fmicb.2026.1825835

**Published:** 2026-06-30

**Authors:** Daria Matyushkina, Anastasia Lazareva, Elena Vasilyeva, Anastasia Polyakova, Alexey Kovalenko, Mikhail Motyakin, Ivan Butenko, Irina Ionova, Maria Sumarokova, Rais Pavlov, Olga Shcherbakova, Maria Tarasova, Fedor Zavalko, Anton Svistunov, Pavel Bashkirov, Vadim Govorun

**Affiliations:** 1 Simple Systems Laboratory, Scientific Research Institute for Systems Biology and Medicine, Rospotrebnadzor, Moscow, Russia; 2 Multi-mix Research Laboratory, Scientific Research Institute for Systems Biology and Medicine, Rospotrebnadzor, Moscow, Russia; 3 Department of Biological and Chemical Physics of Polymers, Emanuel Institute of Biochemical Physics, Russian Academy of Sciences, Moscow, Russia; 4 Semenov Federal Research Center for Chemical Physics, Russian Academy of Sciences, Moscow, Russia; 5 Bioelectrochemistry Laboratory, Scientific Research Institute for Systems Biology and Medicine, Rospotrebnadzor, Moscow, Russia

**Keywords:** glycolysis, metabolism, minimal cell, mycoplasma, sizes, stress adaptation

## Abstract

**Introduction:**

How living cells regulate their function and adapt to stress remains a fundamental question in cell biology. In bacteria, stress responses are classically described at the genomic and transcriptomic levels; however, this paradigm does not adequately explain adaptation in mycoplasmas, which are widely regarded as a model of a minimal cell.

**Methods:**

This study investigated stress adaptation in mycoplasmas by analyzing cellular volume and morphology changes by SEM, DLS, and EPR. Membrane lipid composition was analyzed by mass spectrometry. Intracellular ATP levels were measured using a luciferase‑based assay. Vesicles were isolated by ultracentrifugation and characterized by proteomics, enzyme activity assays, and ATP measurements. Glycolytic flux was assessed in vitro using recombinant enzymes and liposome encapsulation.

**Results:**

Stress adaptation in this minimal cell is primarily achieved through a reduction in cellular volume, mediated by the release of the attachment organelle (“tip”) in the form of vesicles. This process triggers metabolic reorganization within the cell and results in the accumulation of ATP. The metabolic adaptation involves changes in the stoichiometric balance of glycolytic enzymes and alterations in local metabolite concentrations under conditions of reduced cell volume, both of which directly influence glycolytic flux. Concurrently, the released vesicles contain VlhA, lipoproteins, adhesins, hydrogen-peroxide-secreting factors, and exhibit peptidase activity.

**Discussion:**

These findings reveal a novel regulatory mechanism in a minimal cell, where physical volume reduction and biochemical adjustments may be related to an energy-efficient stress response. The dual role of vesicle formation—in cellular remodeling and pathogenesis—deepens our understanding of mycoplasma biology and offers insights for synthetic cell design.

## 1 Introduction

Our understanding of cellular organization and life is largely shaped by the gene-centric central dogma of molecular biology proposed by Watson and Crick in 1958. This framework represented a major conceptual breakthrough, driving the development of molecular biology and enabling the discovery of fundamental regulatory principles in model organisms such as *Escherichia coli* and *Bacillus subtilis*, as well as in more complex eukaryotes. However, in simpler cells such as mycoplasmas, which are widely regarded as a canonical model of a minimal cell, mechanisms of adaptation and regulation cannot be fully explained by this gene-centric paradigm.

Mycoplasmas belong to the Gram-positive bacterial lineage and are characterized by the absence of a cell wall and a markedly reduced genome. This genomic reduction results in a minimal repertoire of transcription factors, approximately an order of magnitude fewer than in *E. coli*. Nevertheless, as demonstrated by Yus et al. in *Mycoplasma pneumoniae*, only about 20% of the transcriptional response to perturbation is mediated by canonical transcription factors (Yus et al., 2019). Instead, the majority of regulation relies on non-canonical mechanisms, including DNA supercoiling, genome organization, and RNA-based processes such as riboswitches, non-coding RNAs, RNA degradation, and the cellular concentration of the initiating nucleoside triphosphate (iNTP).

Even collectively, these mechanisms fail to account for several key features of mycoplasma biology. Notably, transcription and translation become uncoupled under stress (Butenko et al., 2017), many genes are expressed at levels corresponding to fewer than one mRNA transcript per cell (Fisunov et al., 2017), and yet mycoplasmas retain a remarkable capacity to adapt to and survive a wide range of environmental challenges. An extreme example of this resilience is their ability to withstand prolonged nutrient starvation for several weeks despite lacking a sporulation mechanism (Chernov et al., 2009; Demina et al., 2010).

Beyond their value as a model system, mycoplasmas also represent a significant practical concern. They are a common source of cell-line contamination in research laboratories and are responsible for outbreaks in livestock and poultry farming. Moreover, several species are human pathogens, causing pneumonia (Korneenko et al., 2025) and urogenital tract inflammation (Ahmed et al., 2021; Yueyue et al., 2022), with accumulating evidence suggesting potential associations with cancer (Cimolai, 2001; Barykova et al., 2011). This broad ecological and pathogenic diversity is likely linked to their exceptional adaptive capacity, the underlying mechanisms of which remain incompletely understood.

In more complex bacteria, cell size and shape are primarily determined by the cytoskeleton and the cell wall. Beyond these structural constraints, it is well established that growing bacterial cells actively regulate their size and morphology to maintain fitness under stress (Serbanescu et al., 2020). As a result, bacterial populations typically display narrow cell size distributions, with coefficients of variation (CV; standard deviation divided by the mean) that can be as low as 0.1 (Koch, 2006). Using *Salmonella enterica* as a model, Schaechter et al. showed in 1958 that bacterial cell size increases exponentially with population growth rate, a relationship later termed the nutrient growth law (Schaechter et al., 1958). Building on this principle, Serbanescu et al. developed a quantitative model of cell-size regulation in which ribosome abundance serves as a key regulatory variable (Serbanescu et al., 2020). Bacterial cell size depends on ribosomal resource allocation between growth and division, not just growth rate. They showed, that disruption of this balance by antibiotics triggers predictable changes in size and shape, helping cells adapt—for instance, by limiting antibiotic entry or enhancing nutrient uptake.

In contrast, mycoplasmas lack the canonical cytoskeletal elements typical of walled bacteria and are characterized by pronounced pleomorphism, largely attributed to their reduced division and cell wall (DCW) cluster (Martínez-Torró et al., 2021). For several mycoplasmas cytoskeleton is centered on a primitive and highly specialized structure, the terminal organelle (or “tip”), which plays a central role in morphology, cell division, motility, and in attachment to eukaryotic cells. Consequently, variation in cell size among mycoplasmas is unlikely to be as tightly constrained as in bacteria possessing a rigid cell wall. Furthermore, although vesicle secretion has been observed in several mycoplasmas (Gaurivaud et al., 2018; Wang et al., 2024; Wagner et al., 2025), but the mechanism of their formation and, more importantly, their function —particularly with respect to the producing cells—remain poorly understood.

Compared with mycoplasmas, cells with more complex architectures generally attain larger sizes that allow accommodation of the macromolecular machinery required for growth and division. However, increased cell size also reduces the surface-to-volume ratio, thereby decreasing the efficiency of substrate diffusion (Moger-Reischer et al., 2023). For minimal cells such as mycoplasmas, which lack a cell wall and possess limited regulatory networks, diffusion may therefore represent a key mechanism of environmental adaptation. Importantly, diffusion efficiency can be modulated by changes in surface-to-volume ratio, that is, by altering cell size, without reliance on transcriptional regulation.

This principle is illustrated by the synthetically minimized cell *Mycoplasma mycoides* JCVI-syn3B. These cells are smaller than their ancestral strain, and notably, after 2,000 generations of laboratory evolution, the size of the non-minimal control cells increased, whereas the size of the minimal cells remained largely unchanged (Moger-Reischer et al., 2023). These observations suggest that the ability to vary cell size may represent an important mechanism of cellular adaptation.

We therefore propose that maintaining a reduced cell size within a defined range is a necessary condition for the survival of highly reduced bacteria such as mycoplasmas. In this work, we demonstrate for the first time that a universal stress-response strategy in *Mycoplasma gallisepticum* is a reduction in cell size. *In vitro* experiments show that such reduction directly affects the rate of glycolysis, a central energy-producing pathway, suggesting a potential mechanism for rapid metabolic adaptation. Importantly, this response allows mycoplasmas to cope with stress without incurring the time and energetic costs associated with transcriptional and translational regulation.

*M. gallisepticum* achieves cell-size reduction through the formation of extracellular vesicles. By decreasing its volume, the cell adapts its metabolism to changing external conditions through mechanisms that operate independently of genomic or transcriptomic regulation, instead relying on changes in local metabolite concentrations and the stoichiometric balance of glycolytic proteins. This response allows mycoplasmas to cope with stress through a one-time structural remodeling event rather than sustained transcriptional reprogramming. While vesicle formation does entail material costs, these are modest compared to the continuous energy expenditure required for maintaining altered gene expression patterns. We propose that this strategy is energy-efficient in the context of prolonged stress where transcriptional responses would require ongoing ATP investment. At the same time, the vesicles themselves actively contribute to the infectious process. Stress-induced vesicles are enriched in major adhesins, Vlh antigens, peptidases, and NADH oxidase, and they secrete hydrogen peroxide while exhibiting peptidase activity. We hypothesize that these vesicles subsequently interact with host cells, facilitating localized pathogenic effects and bacterial invasion, while their release from the mycoplasma surface enables a concomitant reduction in cell volume.

## 2 Results

### 2.1 *Mycoplasma gallisepticum* decrease cell size under stress

**Figure 1:**
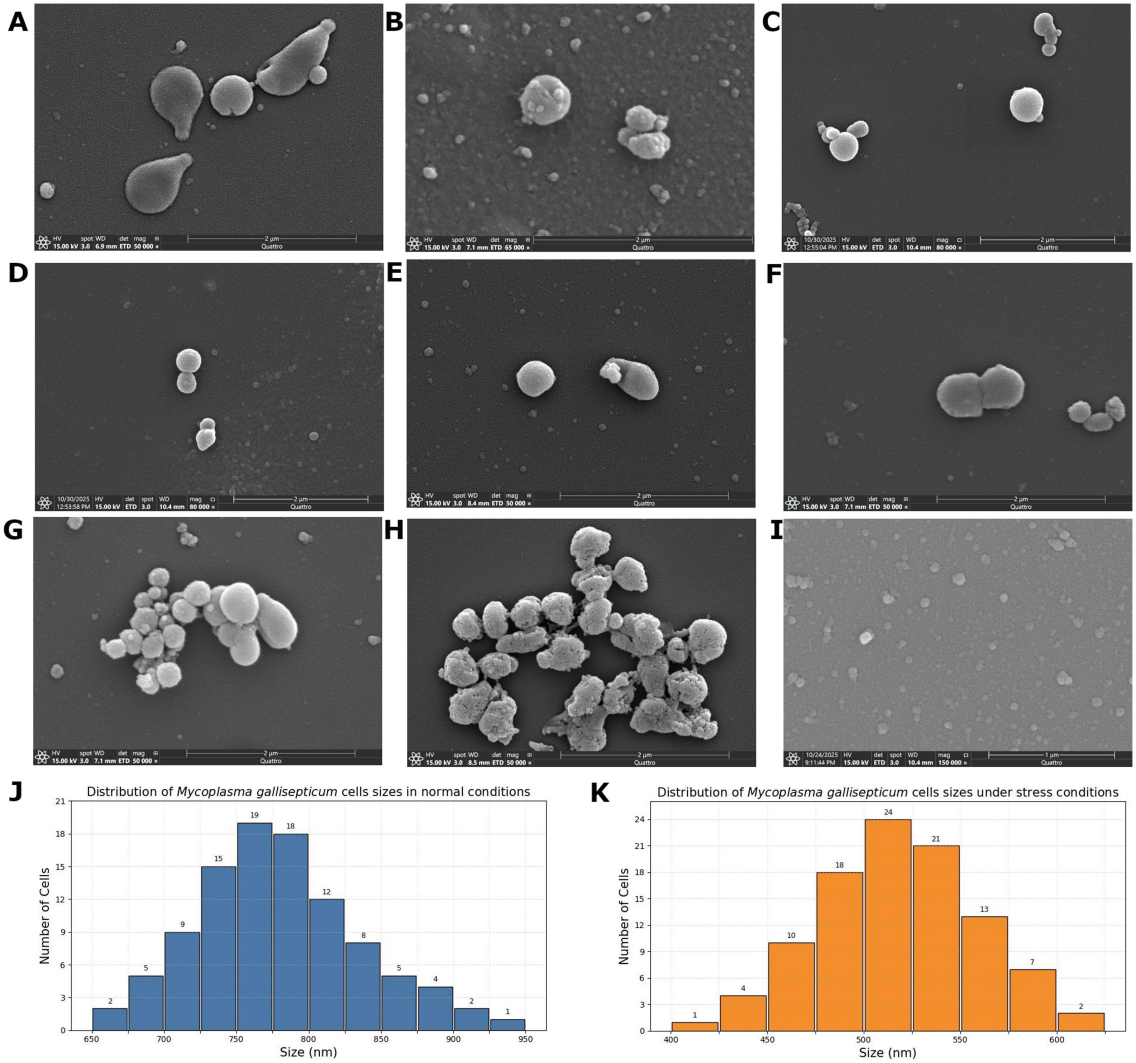
Visualization of *M. gallisepticum* cells and extracellular vesicles under different stress conditions by scanning electron microscopy. **(A)**, control conditions; **(B)**, heat shock (46 °C, 30 min); **(C)**, acute hypoosmotic shock (0 mM NaCl, 30 min); **(D)**, acute hyperosmotic shock (250 mM NaCl, 30 min); **(E)**, chronic hypoosmotic shock (0 mM NaCl); **(F)**, chronic hyperosmotic shock (250 mM NaCl); **(G)**, interaction with host cells (HD3, 24 h of co-cultivation); **(H)**, starvation (nanoforms, 3 weeks of cultivation); **(I)**, extracellular vesicles isolated from *M. gallisepticum* cells. Cell size distribution of *Mycoplasma gallisepticum* under normal **(J)** and stress **(K)** conditions based on scanning electron microscopy results (*n* =100).

We analyzed the size and morphology of *M. gallisepticum* cells using scanning electron microscopy (SEM) under a range of stress conditions (Figure 1). The applied stressors were selected to differ in both their physical impact on the cell and their duration, and included heat shock, acute and chronic osmotic stress, interaction with host cells, and prolonged starvation.

Despite the diversity of these conditions, mycoplasmas exhibited a remarkably consistent response characterized by pronounced changes in cell morphology and size. Specifically, cells transitioned from a pear-shaped morphology to a spherical form, a change that appears to result from the loss of the terminal organelle. Under non-stress conditions, *M. gallisepticum* cells measured approximately 750–900 nm in diameter (Figure 1A). The average cell size was calculated based on measurements of 100 cells (Figure 1J). Assuming a spherical geometry, this corresponds to a cell volume of approximately 0.22–0.38 μm^3^. In addition, a small number of vesicle-like structures with diameters of 100–200 nm was observed on or near the cell surface.

Under stress conditions, cell diameter decreased to approximately 450–600 nm, corresponding to volumes of 0.048–0.113 μm^3^ (Figures 1B–H, K). Overall, stress exposure resulted in an average reduction in mycoplasma cell volume by a factor of 3.38. Notably, during acute stress conditions such as osmotic or heat shock, the majority of cells were substantially smaller, with diameters in the range of 330–360 nm (Figures 1B–D). In addition to cell shrinkage, the presence of swollen cells was also observed under hyperosmotic conditions (Supplementary Figure S1A).

To assess how universal this stress response is across mycoplasmas, we examined *Mycoplasma pneumoniae* cells under normal conditions and during heat shock using SEM (Supplementary Figures S1B,C). Under stress, *M. pneumoniae*, like *M. gallisepticum*, loses its tip organelle, becomes spherical, and decreases in volume.

Across all stress conditions, vesicles morphologically similar to those observed under control conditions were detected, but in markedly higher numbers. To characterize these structures in greater detail, vesicles were isolated by ultracentrifugation and analyzed by SEM (Figure 1I) and atomic force microscopy (AFM) (Supplementary Figure S2). The vesicles exhibited a mean diameter of 108.97 ± 19.33 nm and a mean height of 23.69 ± 8.15 nm.

Scanning electron microscopy inherently introduces potential artifacts related to sample fixation and dehydration. To ensure that the observed changes in cell size and morphology were not caused by sample preparation, we measured the size of living cells using dynamic light scattering (DLS). Measurement parameters were optimized to balance sufficient signal accumulation with the risk of cell heating during acquisition (Supplementary Figure S3).

**Figure 2:**
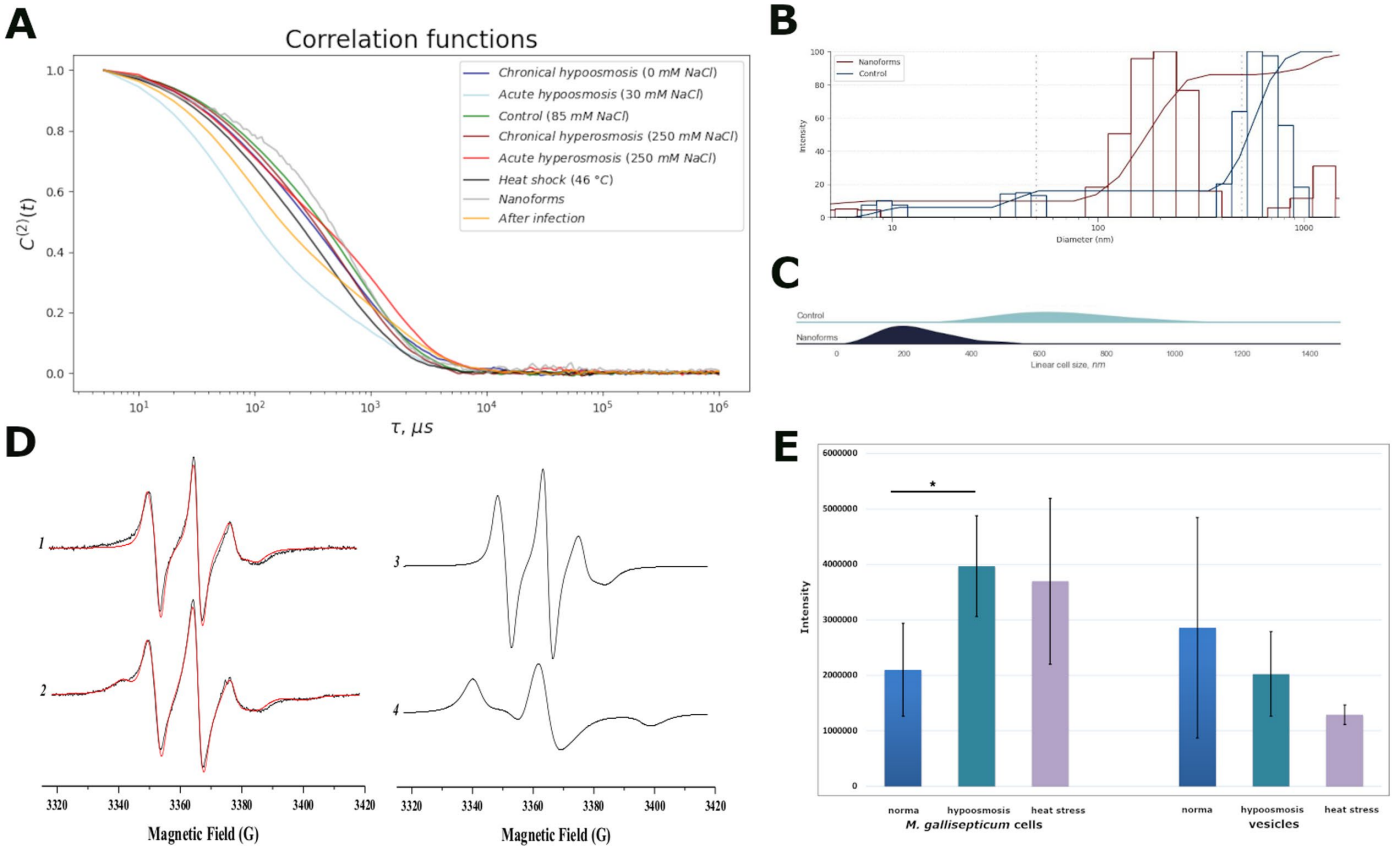
Stress-induced changes in cell size, packing density, and lipid composition of *M. gallisepticum*. **(A)** DLS correlation functions measured for *M. gallisepticum* cells under different stress conditions. For acute hypoosmotic stress experiments, the lowest NaCl concentration used was 30 mM. A lower concentration (e.g., 0 mM) could not be achieved because the protocol required dilution of the original culture with medium containing reduced NaCl concentrations. This approach was chosen to minimize additional cell handling prior to DLS measurements, but it precluded reaching a final NaCl concentration of 0 mM; **(B,C)** Distributions of linear cell sizes for control *M. gallisepticum* cells and under prolonged starvation (nanoforms), determined by DLS analysis. In panel **(C)** conglomerates larger than 1,000 nm were excluded from the particle size distribution histogram; **(D)** Experimental (black line) and theoretical (red line) EPR spectra of the 16-DSA probe in *M. gallisepticum* before (1) and after (2) heat shock. Spectra were recorded at 37 °C. Components 3 and 4 correspond to the spectral contributions of the probe in mycoplasma following heat shock (spectrum 2). After heat shock, the probe spectrum represents a superposition of signals from radicals located in distinct local lipid environments, reflecting the presence of membrane regions with both the baseline packing density observed under control conditions and regions with increased packing density; **(E)** Cholesterol content in *M. gallisepticum* cell membranes and vesicle-enriched fractions under stress versus normal conditions. Asterisks indicate statistically significant differences (*p* < 0.05).

The DLS measurements were largely consistent with the SEM observations. Under all stress conditions, we detected a leftward shift in the correlation function relative to control cells, indicating a reduction in cell size (Figure 2A). An exception was observed under acute hyperosmotic shock, where the correlation function exhibited a slight rightward shift compared to the control. This result agrees with the SEM data, which revealed the presence of a swollen subpopulation of cells under these conditions (Supplementary Figure S2A).

A pronounced rightward shift in the correlation curve was also observed for nanoforms, corresponding to *M. gallisepticum* cells subjected to 3 weeks of starvation, defined by the absence of a carbon source (glucose) as well as purines, pyrimidines, and fatty acids. This shift reflects the presence of cell conglomerates in the culture, as also observed by SEM (Figure 1H), which distort the apparent size distribution (Figure 2B). After excluding these conglomerates from the analysis, the nanoforms exhibited the most substantial reduction in cell size, with an average diameter of approximately 200 nm (Figure 2C). These observations are consistent with previously reported findings (Chernov et al., 2008; Schreiner et al., 2012a).

The stress-induced reduction in *M. gallisepticum* cell size was further supported by electron paramagnetic resonance (EPR) spectroscopy using a spin probe, the nitroxyl radical of 16-doxyl stearic acid (16-DSA). Figure 2D shows the EPR spectrum of 16-DSA in mycoplasma cells under control conditions at 37 °C (spectrum 1, black line). This spectrum is well described within the MOMD framework, yielding a probe rotational correlation time of *τ* = 7.1 × 10^−10^ s and an order parameter S₀ = 0.33 (spectrum 1, red line). Comparable spectra, characterized by similar order parameters and correlation times, have previously been reported for 16-DSA in lipid layers extracted from wheat roots (Calucci et al., 2003).

The isotropic hyperfine coupling (HFC) constant of the probe in mycoplasma lipid layers was *a*_n_ = 14.1 G, a value consistent with that observed for 16-DSA dissolved in hexane (Wasserman et al., 1998). This similarity indicates that the polarity of the lipid environment in which the probe is localized is comparable to that of hexane.

Following heat shock, the EPR spectrum of 16-DSA in *M. gallisepticum* cells (Figure 2D, spectrum 2, black line) could be described as a superposition of two distinct signals arising from radicals located in different local environments. Modeling of the experimental spectrum (Figure 2D, spectrum 2, red line) revealed that the first component (Figure 2D, spectrum 3), accounting for approximately 40% of the total signal, coincides with the spectrum of the probe in lipid layers under control conditions (Figure 2D, spectrum 1). The second component (Figure 2D, spectrum 4), representing approximately 60% of the superposition, is characterized by a markedly slower rotational motion of the radical, with a correlation time of *τ* = 8.3 × 10^−9^ s. This reduced mobility is most plausibly explained by an increase in lipid packing density, corresponding to a decrease in the free volume available for probe rotation within the membrane.

Importantly, the observed response of *M. gallisepticum* cells was rapid, occurring within the ~2-min timeframe required for EPR sample loading and spectral acquisition following heat shock initiation. Heat shock was applied directly within the measurement capillary, and spectra were recorded immediately thereafter. Owing to the constraints of this experimental setup, it was not possible to assess whether similarly rapid changes occur in response to other stress conditions.

Together, these observations indicate that heat shock results in the coexistence of membrane regions with packing densities characteristic of unstressed cells and regions with increased packing density. This increase in membrane density is consistent with the observed reduction in cell size following stress and may additionally contribute to altered cellular regulation by decreasing passive transport across the membrane.

### 2.2 Under stress, the *Mycoplasma gallisepticum* membrane becomes enriched with cholesterol

Analysis of the lipid composition of *M. gallisepticum* cells revealed that stress conditions, including hypoosmotic stress and heat shock, lead to an increase in cholesterol content within the membrane. Figure 2E compares the cholesterol signal intensities of the samples, initially normalized to the copy number of *M. gallisepticum* genomes.

However, if we account for the fact that under stress conditions the average cell volume decreases approximately 3-fold—corresponding to a reduction in cell radius by a factor of ∛3—the cholesterol content can be further adjusted for the change in cell size. Assuming spherical geometry, the surface area (S₂) of stressed cells can be calculated as in Equation 1:


$$S_{2}=4\pi r_{2}^{2}=4\pi {\left(\frac{r_{1}}{\sqrt[{3}]{3}}\right)}^{2}=\frac{4\pi r_{1}^{2}}{{\left(\sqrt[{3}]{3}\right)}^{2}}$$ (1)

Relative to the original surface area (S₁), this yields Equation 2:


$$\frac{S_{2}}{S_{1}}=\frac{1}{{\left(\sqrt[{3}]{3}\right)}^{2}}$$ (2)

Thus, a 3-fold reduction in cell volume results in an approximately 2.08-fold decrease in surface area. When cholesterol content per cell is normalized to account for this reduction in cell size under stress, the cholesterol concentration in the membrane of *M. gallisepticum* is shown to increase substantially. This change is expected to further enhance membrane rigidity and stability, contributing to cellular adaptation and survival under fluctuating environmental conditions. In addition, cholesterol is also identified in vesicles generated by mycoplasma (Figure 2E).

### 2.3 *Mycoplasma gallisepticum* generates vesicles to maintain internal homeostasis

To elucidate the mechanisms underlying vesicle formation, their molecular composition, and the proteomic core retained within the cell following stress-induced size reduction, we performed a comparative proteomic analysis of *M. gallisepticum* cells and their secreted vesicles under heat and osmotic stress conditions. These stressors were selected for all subsequent experiments because their modes of application and the associated sample-preparation procedures allow for the most reliable comparison between stressed and control samples.

**Figure 3:**
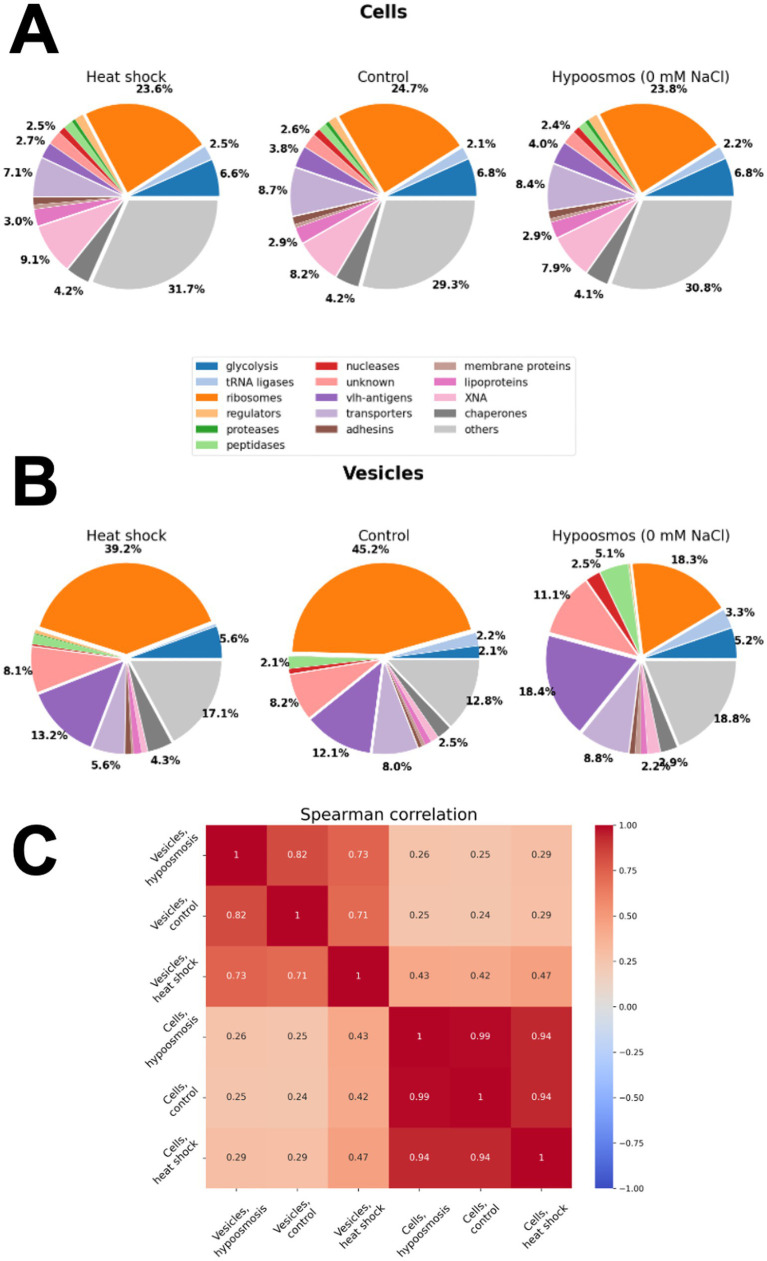
Proteomic changes in *M. gallisepticum* cells and extracellular vesicles under control and stress conditions. **(A)** Distribution of identified proteins by Gene Ontology (GO) categories in cells under control and stress conditions; **(B)** Distribution of identified proteins by GO categories in vesicles under control and stress conditions; **(C)** Heat map showing Spearman correlation coefficients for protein abundance changes in cells and vesicles across conditions.

Analysis revealed that the cellular proteome of *M. gallisepticum* remains largely stable across stress conditions, with no significant changes observed in either functional category representation, as assessed by Gene Ontology (GO) analysis (Figure 3A), or in overall protein abundance (Figure 3C; Supplementary Table S1). In contrast, the proteomic composition of secreted vesicles differed substantially under all examined conditions (Figures 3B,C; Supplementary Table S2). Notably, for many proteins, the direction and magnitude of these changes varied between biological replicates.

We therefore propose that vesicles serve as a flexible means for removing excess or condition-specific molecules, enabling mycoplasma cells to preserve intracellular homeostasis during stress.

### 2.4 Reducing size of *Mycoplasma gallisepticum* leads to an optimization of metabolism

**Figure 4:**
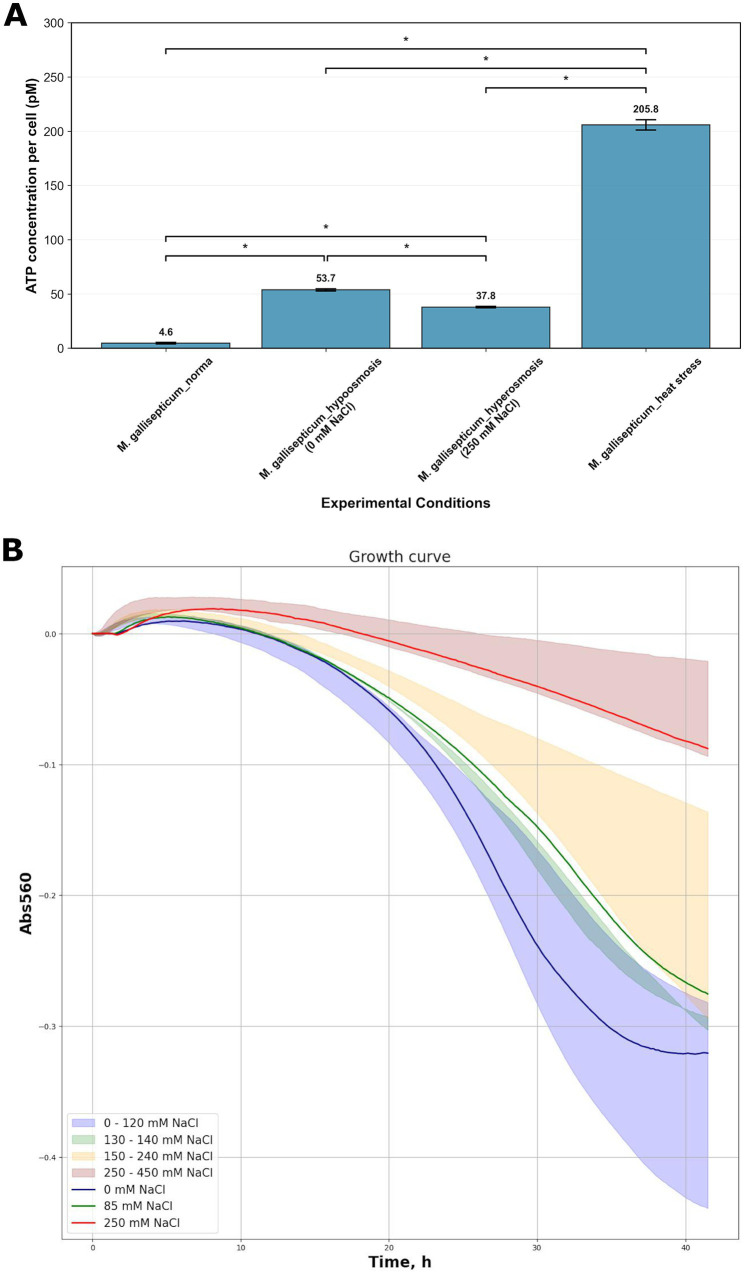
Metabolic activity of *Mycoplasma gallisepticum* cells under different stress conditions. **(A)** Intracellular ATP content in *M. gallisepticum* cells. Asterisks indicate statistically significant differences (*p* < 0.05); **(B)** Metabolic activity curves measured under varying NaCl concentrations in the cultivation medium. Metabolic activity was assessed by monitoring medium acidification using the bromophenol red indicator. The green line denotes control conditions (85 mM NaCl in the culture medium).

We measured intracellular ATP levels in *M. gallisepticum* cells subjected to acute stress (30 min) using a luciferin-luciferase assay (Figure 4A). Under these conditions, we observed a significant accumulation of ATP, most prominently during heat stress, consistent with our previous findings (Butenko et al., 2017). In contrast, ATP levels measured in extracellular vesicles remained stable and did not differ across culture conditions (Supplementary Figure S4).

Based on these observations, we focused on glycolysis as the sole intracellular source of ATP. To assess changes in metabolic activity, we first constructed growth curves in media containing varying NaCl concentrations. Metabolic rate was inferred from the rate of medium acidification during culture growth, as glycolysis in mycoplasmas produces lactate or acetate as end products. Acidification was quantified by monitoring changes in the color of the bromophenol red indicator at an absorbance of 560 nm. Optical density measurements (OD₆₄₀) were not used, as this parameter is strongly influenced by cell size, which varied substantially under our experimental conditions.

Among the stress conditions examined in this study, metabolic activity during growth could be analyzed only under osmotic stress. Cells subjected to heat shock for longer than 1 h did not survive, precluding growth-based measurements. During intracellular infection, mycoplasmas undergo a growth phase in a semi-liquid medium, whereas nanoforms exhibit no detectable metabolic activity. The latter is likely related to the pronounced reduction in cell volume observed for nanoforms, which may correspond to a dormant cellular state.

The range of osmotic conditions permissive for mycoplasma growth extended from 0 to 500 mM NaCl, tested in 10 mM increments. We observed pronounced differences in medium acidification rates across this range (Figure 4B). At NaCl concentrations between 0 and 120 mM, cells acidified the medium more rapidly than under control conditions (85 mM NaCl, black line). Concentrations of 130–140 mM corresponded to near-normal metabolic activity. At NaCl concentrations between 150 and 240 mM, medium acidification occurred more slowly than in the control. A sharp decline in metabolic activity was observed at 250 mM NaCl, likely reflecting cell swelling and subsequent lysis under hyperosmotic conditions, as detected by SEM. Under these circumstances, reliable estimation of per-cell metabolic activity becomes difficult. At higher NaCl concentrations (300–500 mM), cell growth was no longer detectable.

It is noteworthy that in natural environments mycoplasmas primarily inhabit intracellular niches, where NaCl concentrations are low. Consequently, these organisms are expected to be better adapted to hypoosmotic conditions.

Because mycoplasma cell volume decreases under stress, intracellular concentrations of metabolites are expected to increase. Given that several metabolites have been shown to inhibit glycolytic reactions (Turner and Plaxton, 2000; Maeda et al., 2003; Feksa et al., 2005), we next examined how the addition of such metabolites to the culture medium affects *M. gallisepticum* metabolism under both normal and hypoosmotic conditions, the latter being associated with reduced cell volume.

Addition of the tested metabolites resulted in a concentration-dependent reduction in mycoplasma growth. This inhibitory effect was significantly more pronounced under hypoosmotic conditions, where cells exhibited marked volume reduction (Supplementary Figure S5).

### 2.5 *Mycoplasma gallisepticum* controls its metabolism through metabolites concentration and glycolytic proteins stoichiometry

**Figure 5:**
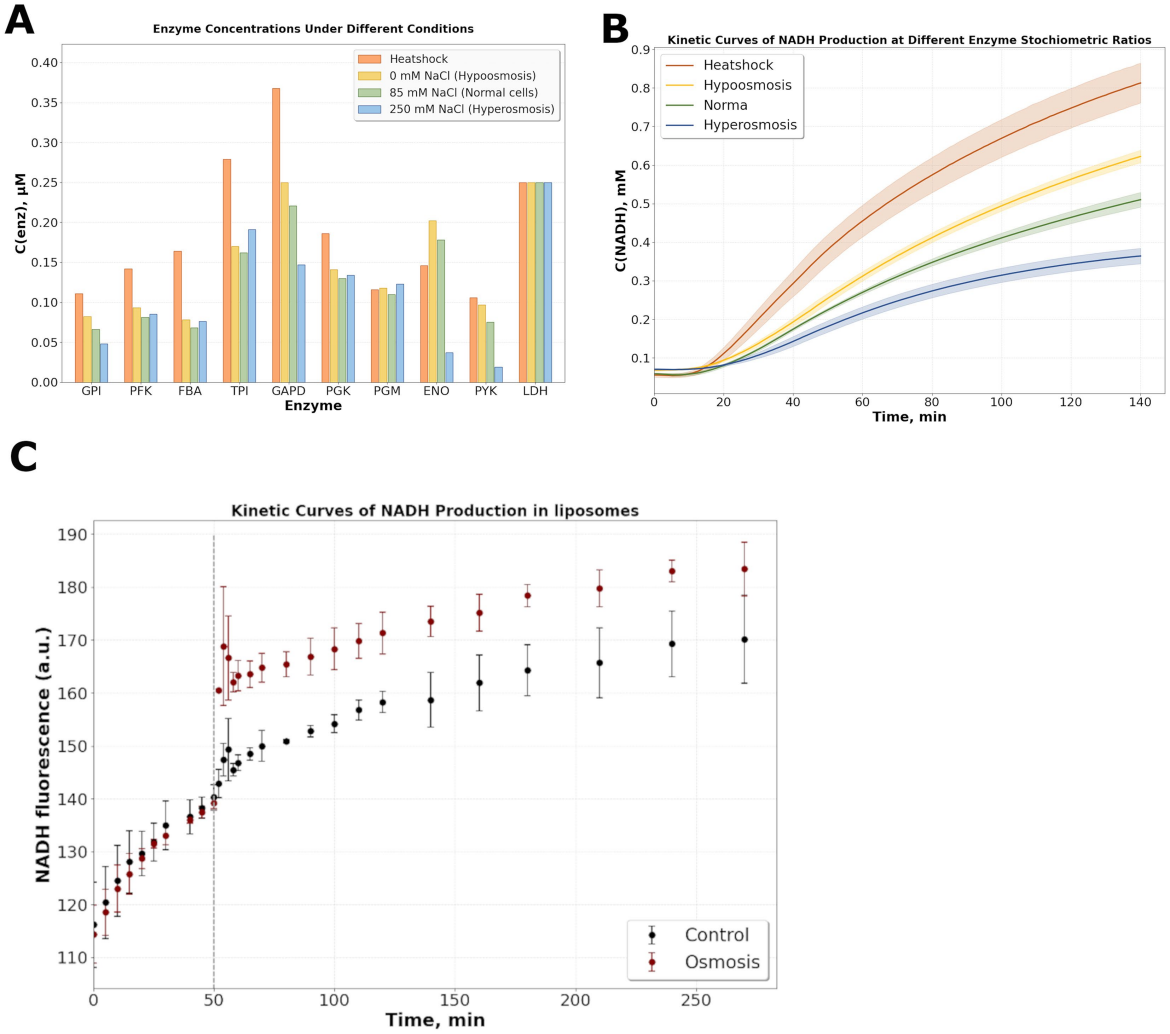
Influence of glycolytic enzyme stoichiometry on glycolytic activity in *M. gallisepticum* under different stress conditions. **(A)** Relative stoichiometric ratios of glycolytic proteins in *M. gallisepticum* cells under control and stress conditions, determined by mass spectrometry; **(B)** *In vitro* kinetic curves of NADH production measured at a fixed ATP concentration of 5 mM using enzyme stoichiometric ratios derived from *in vivo* measurements; **(C)** Real-time monitoring of glycolysis kinetics via NADH fluorescence in N-Lip liposomes. Effect of hyperosmotic stress applied after 50 min of incubation under isoosmotic conditions (red), compared to the uninterrupted isoosmotic control (grey).

Although our proteomic analysis of *M. gallisepticum* under different stress conditions indicated that the overall protein composition remains largely unchanged, we specifically examined stress-induced changes in the stoichiometric ratios of glycolytic enzymes. We reasoned that, under conditions of reduced cell volume, even modest shifts in these ratios could substantially influence the glycolytic rate. Protein abundances were normalized to lactate dehydrogenase, and the most pronounced deviations from control stoichiometry were observed under heat shock conditions (Figure 5A).

To directly assess the impact of altered glycolytic enzyme ratios on reaction kinetics, we performed *in vitro* reconstitution experiments. Recombinant glycolytic proteins from *M. gallisepticum* were combined in solution at the relative ratios determined experimentally (Figure 5A), and the glycolytic reaction rate was quantified by monitoring the accumulation of NADH (Figure 5B). Even under these simplified conditions, which do not account for changes in cellular volume, the glycolytic reaction rate was higher for enzyme ratios corresponding to heat shock and hypoosmotic stress than for those observed under control conditions. In contrast, glycolysis was reduced when enzyme ratios corresponding to hyperosmotic stress were used.

This reduction may reflect the lower tolerance of *M. gallisepticum* to hyperosmotic conditions, as indicated by SEM and DLS analyses, which revealed a subpopulation of swollen cells under these conditions. During proteomic analysis, it was not possible to separate swollen cells from the remainder of the population. Consequently, the measured stoichiometric ratios of glycolytic proteins under hyperosmotic stress represent population averages across cells of differing sizes. As a result, the *in vitro* kinetic reconstruction for these conditions may not fully capture the true intracellular state of individual cells.

Given the observed accumulation of ATP in *M. gallisepticum* cells under stress (Figure 4A), together with the concentration-dependent inhibition of growth by extracellular ATP (Supplementary Figure S5C), we examined the effect of ATP on *in vitro* glycolytic kinetics (Supplementary Figure S6). Across all stress-related enzyme stoichiometries, increasing ATP concentrations resulted in a progressive slowdown of glycolysis.

Furthermore, we analyzed glycolytic kinetics under volume reduction conditions by simulating the response of *M. gallisepticum* cells to osmotic stress. To this end, recombinant glycolytic enzymes were encapsulated in liposomes. The assessment of liposome compression under hyperosmotic conditions was analyzed by calcein fluorescence quenching.

Kinetic evaluation of reconstituted glycolysis in liposomes consisting of POPC, DOPS, and Liss Rhod PE (N-Lip liposomes) revealed steady NADH production over 4 h, confirming the functional operation of the encapsulated pathway (Figure 5C, grey circles). The characteristic reaction time under isoosmotic conditions (τ_control_), calculated using Equation 5 (see Materials and methods), was 77 ± 5 min.

Application of hyperosmotic stress induced a rapid increase in NADH fluorescence intensity (up to 13%; Figure 5C, red circles). This phenomenon can be attributed to a momentary acceleration of NADH production immediately following osmotic shock, likely due to a rapid concentration of internal reactants and enzymes as the liposomes shrink. Within minutes, the system re-established equilibrium, and the overall kinetic parameters stabilized.

### 2.6 Vesicles as a pathogenicity factor

We next examined whether vesicles are not merely a byproduct of stress-induced cell size reduction, but also play an active role in the infectious process. Although the proteomic composition of vesicles is highly variable (Figures 3B,C), the enrichment dynamics of adhesins, VlhA antigens, and lipoproteins suggest that vesicle formation is initiated preferentially in the region of the terminal (“tip”) organelle, particularly under stress conditions. This interpretation is further supported by SEM micrographs (Figure 1; Supplementary Figure S1A).

Mass spectrometry analysis indicates that vesicles contain nearly the entire VlhA protein, rather than only its C-terminal domain (Supplementary Figure S7). Thus, by shedding surface antigens into vesicles, mycoplasmas may evade host immune recognition. In addition, because vesicles carry adhesins and antigenic determinants on their surface, they are likely capable of binding to eukaryotic cells and exerting pathogenic effects independently of intact bacterial cells.

**Figure 6:**
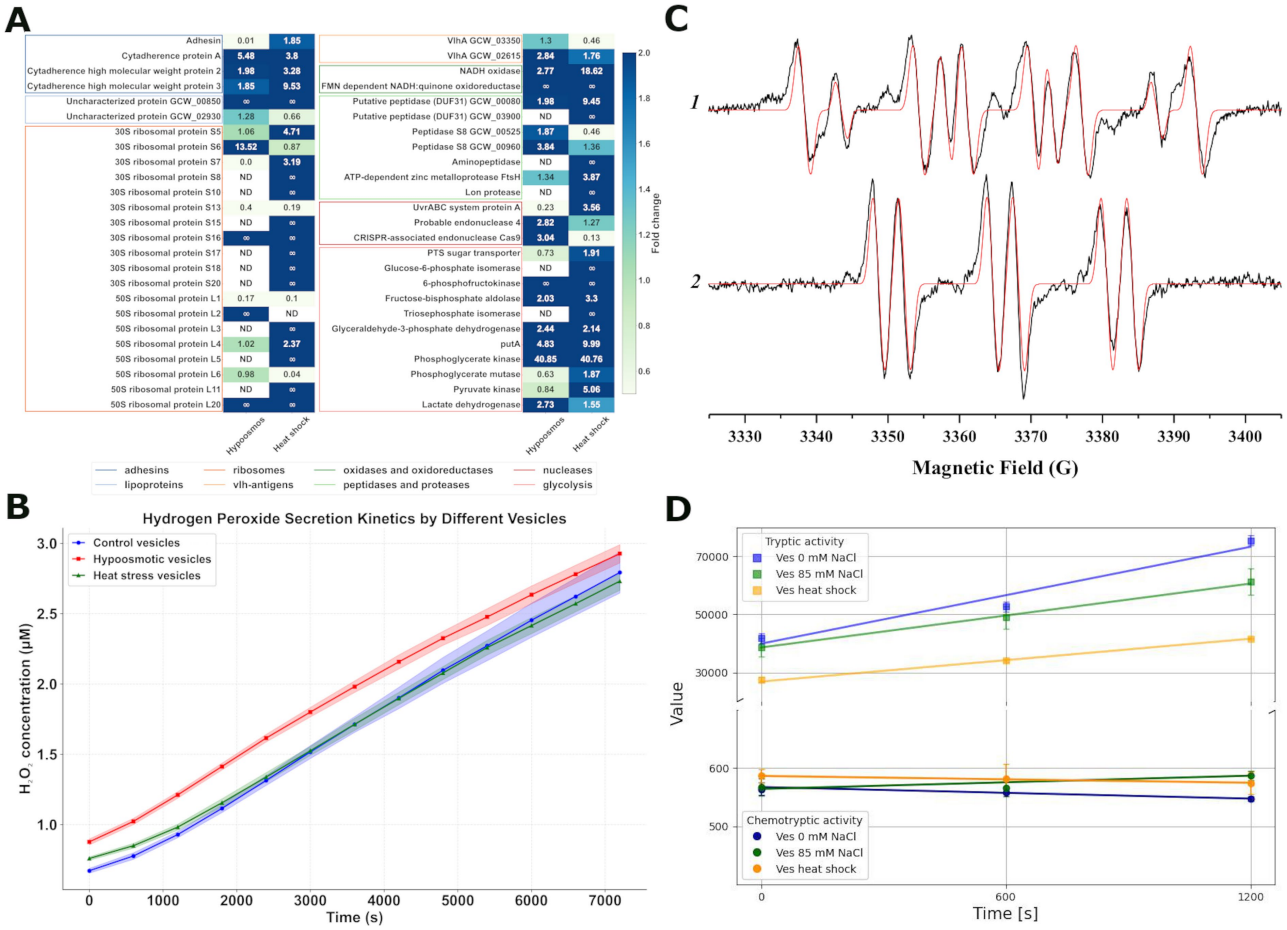
Composition and functional activity of extracellular vesicles derived from *M. gallisepticum* under stress conditions. **(A)** Protein groups showing the most pronounced changes in representation in vesicles isolated from stressed *M. gallisepticum* cells. **(B)** Hydrogen peroxide (H₂O₂) production by *M. gallisepticum* vesicles; **(C)** Reactive oxygen species (ROS) production under *M. gallisepticum* heat stress conditions. Experimental (black line) and simulated (red line) EPR spectra of spin-trap adducts formed with DMPO (1) and PBN (2); **(D)** Peptidase activity of *M. gallisepticum* vesicles assessed using fluorogenic peptide substrates.

We further investigated the ability of vesicles to produce hydrogen peroxide, a major nonspecific factor contributing to mycoplasma pathogenicity. Proteomic analysis revealed the presence of NADH oxidase in vesicles, with increased abundance under stress conditions (Figure 6A). We therefore directly measured hydrogen peroxide production by vesicles (Figure 6B). In addition, stress-induced vesicles showed upregulation of FMN-dependent NADH:quinone oxidoreductase (AzoR) (Figure 6A).

To further support the production of reactive oxygen species, we used spin-trap electron paramagnetic resonance (EPR) spectroscopy with the DMPO spin trap and demonstrated the formation of hydroxyl (•OH) radicals under heat shock conditions. Figure 6C shows the EPR spectrum of DMPO spin adducts in *M. gallisepticum* subjected to heat shock (spectrum 1, black line). This spectrum is well described by a superposition of signals from two distinct nitroxyl radicals (spectrum 1, red line) with hyperfine coupling (HFC) constants of a_n_ = 14.8 G and a_h_ = 14.7 G (line intensity ratio 1:2:2:1), and a_h_ = 16.0 G and a_h_ = 22.8 G. The parameters of the first radical closely match reported HFC constants for the DMPO adduct formed upon trapping hydroxyl radicals (Buettner, 1987). Notably, similar parameter values have also been reported for DMPO adducts formed with lipid alkoxy radicals (Schaich and Borg, 1990).

The HFC constants of the second signal are indicative of a carbon-centered radical (Buettner, 1987). Although the precise structure of this radical cannot be conclusively identified, the measured magnetic parameters closely resemble those reported for carbon-centered radicals generated during lipid oxidation (a_n_ = 16.1 G, a_h_ = 23.3 G) (Schaich and Borg, 1990; Lipid Oxidation, Antioxidants, and Spin Trapping, 2007). To further validate the presence of carbon-centered radicals, we employed an additional spin trap, PBN. The EPR spectrum of PBN adducts formed in *M. gallisepticum* under heat shock conditions appeared as a triplet of doublets with HFC constants a_n_ = 15.9 G and a_h_ = 3.65 G (Figure 6C, spectrum 2), values characteristic of carbon-centered radicals (Buettner, 1987). Comparable parameters have been reported for radicals generated during lipid oxidation (Sata et al., 1990; Novakov et al., 2001), and potential radical structures arising from lipid oxidation have been discussed previously (Foret et al., 2020). Together, these data indicate that hydroxyl radicals are generated in *M. gallisepticum* under heat shock and likely initiate lipid oxidation processes.

Proteomic analysis further revealed that stress-induced vesicles are enriched in peptidases (Figure 6A), including two putative DUF31 domain-containing peptidases (GCW_00080 and GCW_03965), as well as additional DUF31-containing proteins (GCW_03070 and GCW_01705). In addition, stress vesicles contain the ATP-dependent proteases Lon and FtsH (Figure 6A), which may participate in the vesiculation process, although this possibility requires further investigation. Consistent with the proteomic data, we detected trypsin-like peptidase activity in vesicles using fluorometric assays (Figure 6D). Our analysis of vesicle content also revealed the presence of ribosomal proteins from both the small and large ribosomal subunits (Figure 6A).

## 3 Discussion

In 1962, Nobel laureates François Jacob and Jacques Monod described the structure and regulation of the *Escherichia coli* lac operon, establishing a canonical framework for gene regulation that has since been broadly applied across biology (Jacob and Monod, 1961). In this model, gene expression proceeds through transcription of DNA into mRNA, followed by translation of mRNA into protein. The coordination of transcriptional and translational fluxes, together with the predominance of transcriptional control, led to a quantitative formulation of the Central Dogma in bacteria (Balakrishnan et al., 2022). Although this framework has been refined over the decades, its core principles have remained largely unchanged. However, an increasing body of evidence now indicates that many regulatory phenomena in bacteria cannot be fully explained by a strictly gene-centric model. This limitation is particularly evident in bacteria with reduced genomes and a minimal repertoire of transcription factors, such as members of the genus *Mycoplasma*, which serve as valuable model systems in systems and synthetic biology.

Despite their minimal genomes, mycoplasmas remain highly complex biological systems. Owing to the loss of many canonical regulatory pathways present in bacteria such as *E. coli*, these organisms face significant challenges in maintaining metabolic homeostasis, particularly under stress conditions. It is therefore plausible that mycoplasmas rely on a single integrative mechanism capable of simultaneously addressing multiple regulatory demands. Our results support such a model, demonstrating that stress-induced reduction in cell size, coupled with vesicle formation, constitutes a central adaptive strategy. This response enables rapid metabolic adjustment to changing environmental conditions, facilitates evasion of host immune defenses, and promotes the progression of infection.

Using scanning electron microscopy (SEM) (Figure 1; Supplementary Figure S1A) and dynamic light scattering (DLS) (Figure 2A), we observed a consistent reduction in *M. gallisepticum* cell size across a range of stress conditions, including heat shock, hypo- and hyperosmotic stress, starvation, and interaction with host cells. On average, cell volume decreased by a factor of 3.38. Interestingly, a similar effect was also observed in another species, *Mycoplasma pneumoniae* (Supplementary Figures S1B,C), suggesting that this response may be universal. And such cell reaction is likely enabled by the unique structural features of mycoplasmas, which lack a rigid cell wall and therefore exhibit pronounced pleomorphism.

In addition to cell shrinkage, osmotic stress, particularly under hyperosmotic conditions, was also associated with the presence of swollen cells (Supplementary Figure S1A). This observation is consistent with previous studies showing that, in the absence of glucose, *M. gallisepticum* cells incubated in an isosmotic NaCl solution (≈250 mM) display reduced adenosine triphosphate (ATP) levels and proton motive force (ΔμH^+^), along with an increased tendency to swell and lyse compared to glucose-supplemented cells. These findings indicate that cellular energy is required for proper regulation of cell volume (Rottem et al., 1981; Linker and Wilson, 1985).

Concomitant with cell size reduction, *M. gallisepticum* undergoes substantial remodeling of its membrane lipid composition by enrichment of the membrane through the enrichment of cholesterol (Figure 2E). This enrichment is expected to reduce membrane fluidity and enhance rigidity, potentially providing additional protection under stress conditions. Consistent with these compositional changes, increased membrane packing density following stress was independently confirmed using spin-probe EPR spectroscopy (Figure 2D).

Despite their pronounced pleomorphism, several mycoplasma species exhibit a well-defined cell morphology. In particular, *M. pneumoniae*, *M. genitalium*, and *M. gallisepticum* display a characteristic pear-shaped form with a terminal (“tip”) organelle located at the narrow end of the cell. This organelle is a complex structure composed of numerous cytoskeletal proteins, heat shock proteins, enzymes of energy metabolism, and the majority of cytadherence-related proteins (Yueyue et al., 2022). Numerous studies have reported the presence of glycolytic enzymes on the mycoplasma cell membrane (Yavlovich et al., 2007; Chen et al., 2011; Schreiner et al., 2012b; Thomas et al., 2013; Bao et al., 2014; Gründel et al., 2016); however, the functional significance of this localization remains incompletely understood. Most explanations have focused on proposed secondary roles of these enzymes as adhesins.

Notably, the subcellular fraction containing the DNA replication complex in *M. gallisepticum* has been shown to co-localize with the terminal organelle, suggesting a physical association between chromosomal DNA and the attachment structure. Based on these observations, Quinlan and Maniloff proposed that the tip organelle plays a role in organizing DNA replication in this organism (Quinlan and Maniloff, 1972; Pich et al., 2009).

In the present study, we observed that *M. gallisepticum* and also *M. pneumoniae* cells respond to diverse stress conditions by reducing cell size through the release of the tip organelle via vesicle formation, accompanied by a morphological transition from a pear-shaped to a spherical form (Figure 1; Supplementary Figure S1A). Vesicles forming consistent with previous reports for other mycoplasma species (Gaurivaud et al., 2018). Such large-scale structural remodeling may influence nucleoid organization and could therefore represent an additional layer of regulatory control in mycoplasmas.

Consistent with this model, adhesins, VlhA antigens, and lipoproteins, which are normally concentrated within the tip region, were abundantly detected in stress-induced vesicles (Figure 6A; Supplementary Table S2). Moreover, the shedding of VlhA proteins from the bacterial surface into vesicles effectively removes major antigenic determinants from the cell envelope. This is consistent with our previously obtained data that *M. gallisepticum* cells lose hemagglutinating activity following heat shock (Butenko et al., 2017). This process likely renders mycoplasma cells less accessible to antibody recognition, thereby facilitating immune evasion and enabling long-term persistent infection.

*Mycoplasma gallisepticum* also produces extracellular vesicles under non-stress conditions. However, in control cultures we did not observe a complete morphological transition from pear-shaped to spherical cells (Figure 1A), and vesicles formed under these conditions contained substantially lower levels of proteins typically localized to the terminal (“tip”) region (Figure 6A; Supplementary Table S2). These observations suggest that vesicle formation under normal and stress conditions proceeds via distinct mechanisms.

Across all stress conditions examined, the proteomic composition of *M. gallisepticum* cells themselves remained largely unchanged (Figure 3A; Supplementary Table S1), which is consistent with previously reported findings (Butenko et al., 2017), indicating that the cell actively maintains intracellular proteome stability. Vesicle formation may therefore serve as a mechanism for preserving intracellular homeostasis through the selective removal of excess or context-dependent components.

Under both hypo- and hyperosmotic conditions, at sublethal NaCl concentrations, *M. gallisepticum* exhibited increased metabolic activity (Figure 4B). Although the overall proteome remained stable, stress-induced reductions in cell volume are expected to cause local increases in intracellular metabolite concentrations, which can substantially influence metabolic flux. Previous studies have shown that elevated concentrations of certain metabolites can inhibit glycolytic reactions (Turner and Plaxton, 2000; Maeda et al., 2003; Feksa et al., 2005). Consistent with this, we found that supplementation of the culture medium with ATP, ADP, CTP, glucose-6-phosphate, glyceraldehyde-3-phosphate, or tryptophan resulted in a dose-dependent reduction in *M. gallisepticum* metabolic activity (Supplementary Figure S5). Notably, this inhibitory effect was significantly more pronounced in cells that had undergone stress-induced size reduction.

Because glycolysis represents the central metabolic pathway in mycoplasmas and serves as the primary source of intracellular ATP, we analyzed stress-induced changes in the stoichiometric ratios of glycolytic enzymes in *M. gallisepticum* and quantified intracellular ATP levels (Figure 4A). Under all stress conditions examined, ATP accumulation was observed, with the most pronounced increase occurring during heat shock. The more modest ATP increase detected under osmotic stress may reflect active Na^+^ extrusion via a primary transport mechanism, which *M. gallisepticum* employs for cell volume regulation (Shirvan et al., 1989). In addition, surface attachment and motility in mycoplasmas, both mediated by the terminal (“tip”) organelle, are ATP-dependent processes (Chen et al., 2025). Consequently, the stress-induced loss of the tip organelle observed here may further contribute to intracellular ATP accumulation by reducing ATP consumption.

Given that the stoichiometric ratios of glycolytic enzymes varied substantially across stress conditions (Figure 5A), we next examined how these changes affect glycolytic reaction kinetics *in vitro*. Analysis of the kinetic curves demonstrated that enzyme ratios corresponding to heat shock and hypoosmotic stress conditions led to increased glycolytic rates (Figure 5B). The increase in glycolytic rate is also supported by experiments involving the encapsulation of glycolytic proteins in liposomes. Under hyperosmotic conditions, liposome compression induces a sharp spike in NADH levels (Figure 5C). At the same time, elevating ATP concentrations in the reaction mixture resulted in a slowdown of glycolytic kinetics (Supplementary Figure S6). *In vivo*, this combination of accelerated glycolysis and ATP-mediated feedback inhibition could promote temporary metabolic arrest and thereby enabling mycoplasma cells to adapt to fluctuating environmental conditions, or, under extreme conditions, the formation of a dormant state. We acknowledge that our *in vitro* glycolytic reconstitution lacks native membrane association, macromolecular crowding, and physiological metabolite ratios. These experiments should be interpreted as proof-of-principle that stoichiometric changes alone can alter flux, not as quantitative predictions of *in vivo* rates.

Thus, we conclude that under stress conditions mycoplasmas reduce cell size by shedding the terminal (“tip”) organelle in the form of extracellular vesicles. This process enables *M. gallisepticum* to rapidly and energy-efficiently adapt its metabolism to changing environmental conditions without engaging gene regulatory networks or the translational machinery, both of which are energetically costly and kinetically slow. The resulting accumulation of ATP can therefore be redirected toward essential stress-response processes, enhancing cellular survival under adverse conditions.

An important question arising from our findings is whether vesicles secreted by *M. gallisepticum* play an active role in the infectious process. Our proteomic analysis revealed stress-induced enrichment of the FMN-dependent NADH:quinone oxidoreductase (AzoR) in vesicles following heat shock (Figure 6A; Supplementary Table S1). Quinone reductases have been reported to contribute to hydrogen peroxide resistance (Gonzalez et al., 2005; Hong et al., 2008) and to play roles in host colonization (Wang and Maier, 2004), further supporting the involvement of vesicles in pathogenic interactions. Nicotinamide adenine dinucleotide-dependent flavin oxidoreductases have previously been shown to localize to the surface of *Mycoplasma hyopneumoniae* cells and to function as potential virulence factors (Xie et al., 2021), with similar observations reported for *Mycoplasma synoviae* (Hu et al., 2022).

In addition to AzoR, vesicles were found to contain glycolytic enzymes and NADH oxidase, which catalyzes the conversion of pyruvate to acetate with concomitant production of hydrogen peroxide (Figure 6A; Supplementary Table S1). Consistent with this pathway, Consistent with this, we demonstrated that vesicles are capable of secreting H₂O₂ in the presence of glucose (Figure 6B). The generation of reactive oxygen species and the occurrence of lipid peroxidation were further confirmed by EPR spectroscopy using spin traps (Figure 6C). Hydrogen peroxide is known to exert damaging effects on host cell membranes, which may facilitate mycoplasma penetration into host cells, particularly given that many mycoplasmas exhibit an intracellular lifestyle. It is also possible that H₂O₂ production contributes to vesicle formation itself, although this hypothesis requires further investigation.

Stress-induced vesicles were additionally enriched in DUF31 domain-containing peptidases, which have been associated with mycoplasma pathogenicity (Arfi et al., 2016). It has been proposed that the immunoglobulin-binding protein M functions in concert with this domain as part of the MIB–MIP system, a recently described mechanism that protects mycoplasmas from host Ig-mediated immune responses (Qin et al., 2019). In agreement with these observations, we detected trypsin-like peptidase activity in vesicles (Figure 6D). Such activity may contribute to post-translational processing of surface proteins and facilitate interactions with host cells, thereby enhancing bacterial entry.

According to the model of cell size regulation proposed by Serbanescu et al. (2020), a reduction in ribosome abundance is associated with a decrease in cellular volume. This relationship may explain the redistribution of ribosomal proteins into vesicles under stress conditions observed here (Figure 6A). It should be noted, however, that in contrast to the experiments by Serbanescu et al., our culture conditions were not nutrient-enriched. Moreover, Lon protease has been implicated in ribosome stalling (Burgos et al., 2021). Consequently, the co-occurrence of Lon, ribosomal components, and other proteins within vesicles may reflect the degradation of tmRNA-tagged translation products taking place inside vesicles.

At present, no single universal mechanism has been identified for mycoplasma penetration into host cells. Several invasive mycoplasma species are known to induce cytoskeletal rearrangements following host-cell interaction, enabling internalization through actin microfilament- and microtubule-dependent pathways (Xiu et al., 2024). Based on our results, we propose that mycoplasma-derived vesicles represent an initial line of attack on host cells. By carrying adhesins, lipoproteins, and enzymatic activities on their surface, these vesicles may directly interact with host cell membranes, creating favorable conditions for subsequent mycoplasma entry and infection.

We do not propose active sorting of cytosolic contents into vesicles. Rather, the tip organelle—a structurally distinct cellular compartment with defined protein composition—is shed as vesicles. This removes surface-exposed proteins and tip-associated enzymes while leaving the cytosol intact. The subsequent reduction in cell volume passively concentrates remaining intracellular metabolites and cytosolic proteins, altering metabolic flux without requiring selective sorting mechanisms.

Thus, our study demonstrates that *M. gallisepticum* employs a universal stress-adaptation strategy based on the reduction of cell volume. This mechanism enables a rapid response that bypasses regulation at the genomic and transcriptional levels and does not require activation of the translational machinery, a phenomenon previously observed but not fully explained (Maier et al., 2011; Butenko et al., 2017; Miravet-Verde et al., 2025). Instead, metabolic adaptation to stress is achieved through localized changes in metabolite concentrations (“push” mechanism) and through adjustments in the stoichiometric balance of glycolytic enzymes (“pull” mechanism)—a process that is intrinsically energy-efficient. We propose that, in mycoplasmas, the passive concentration mechanism (“push”) may dominate during acute stress, while stoichiometric adjustments (“pull”) become more relevant during chronic adaptation. As a result, cellular energy is conserved, providing a reserve that supports bacterial survival under altered environmental conditions.

Notably, stress-induced cell size reduction is not unique to mycoplasmas. In industrial bioprocesses, microorganisms exposed to osmotic stress, nutrient limitation, or mechanical shear frequently exhibit decreased cell volume, often accompanied by altered metabolic productivity (Stewart and Robertson, 1989; Kataoka et al., 2022). While those studies typically interpret such changes within the framework of gene regulation and signal transduction, our findings suggest that a purely physicochemical mechanism—passive concentration of metabolites—may contribute independently. We propose that volume reduction represents a universal, energy-efficient stress response that operates in parallel with canonical regulatory networks, and may be particularly important in organisms with reduced genomes or under conditions where transcriptional responses are too slow or costly.

## 4 Materials and methods

### 4.1 Cell culturing and stress conditions

*M. gallisepticum* S6 cells were cultivated, and nanoforms were obtained as described previously (Demina et al., 2010). Osmotic stress conditions were generated by adjusting the NaCl concentration in the cultivation medium. Hypoosmotic conditions were achieved by omitting NaCl from the medium, although trace amounts remained due to the presence of yeast dialysate and horse serum. Hyperosmotic conditions were established by increasing the NaCl concentration to 250 mM, compared with the standard concentration of 85 mM.

For acute osmotic stress, mycoplasma cells were grown to the mid-logarithmic phase, collected by centrifugation, and resuspended in fresh medium containing the desired NaCl concentration. Cells were then incubated at 37 °C for 30 min. For chronic osmotic stress, cells were directly subcultured into media with the indicated NaCl concentrations and grown at 37 °C until reaching the mid-logarithmic phase. Heat shock was induced by incubating cells at 46 °C for 30 min. Conditions for obtaining *M. gallisepticum* following intracellular localization within HD3 cells were as described previously (Matyushkina et al., 2016). Co-incubation of *M. gallisepticum* with HD3 cells was carried out for 24 h.

The *M. pneumoniae* strain (clinical isolate) was a kind gift from the Centre for Strategic Planning and Management of Biomedical Health Risks (Moscow, Russia). The bacteria were cultured in tissue culture flasks (25 cm^2^) until confluent, but before the medium acidified, at 37 °C and 5% CO₂ in medium (pH 7.8) containing the following components: PPLO (Himedia, India) 18.5 g/L; yeast extract (Laboratorios Conda, Spain) 1.5 g/L; phenol red; glucose 1%; Medium 199 (NPP PanEco, Russia) 15%; fetal bovine blood serum (NPP PanEco, Russia), pre-inactivated by heating at 56 °C for 30 min, 20%; and ampicillin sodium salt (Solarbio, China) 500 mg/L. Heat shock was performed on the *M. pneumoniae* culture at 42 °C for 30 min.

### 4.2 Bacterial growth curves

Mycoplasma growth curves were evaluated by measuring cellular metabolic activity, assessed through acidification of the culture medium. Medium acidification was monitored using the bromophenol red pH indicator, and absorbance was recorded at 560 nm (Abs₅₆₀) with a Tecan Infinite® M Plex Infinite 200 Pro multimode plate reader. Measurements were acquired every 15 min at 37 °C using a 96-well plate format.

To examine the effect of metabolites on the growth rate of *M. gallisepticum* under normal and hypoosmotic conditions, defined concentrations of individual metabolites (tryptophan, ATP, ADP, CTP, glucose-6-phosphate, or glyceraldehyde-3-phosphate) were added to the culture medium. Experiments were performed in media containing either standard NaCl concentration (85 mM; control conditions) or no added NaCl (0 mM; hypoosmotic conditions).

### 4.3 Cell size determination by dynamic light scattering

Dynamic light scattering (DLS) analyses were performed using a 90Plus Nanoparticle Size Analyzer equipped with a Peltier temperature control system (Brookhaven Instruments Corp., Holtsville, NY). Cell size measurements were carried out at a fixed scattering angle of 90° and a wavelength of 661 nm.

To maximize measurement accuracy, cells were diluted prior to DLS analysis using a medium appropriate for the specific experimental condition. Sample concentrations were adjusted according to the manufacturer’s recommendation, such that the detected signal intensity ranged between 700 and 900 kilocounts per second (kcps). Automatic exclusion of large scattering events was enabled using the instrument’s “Dust Filter” function. Measurements were conducted at 25 °C.

For each experimental condition, ten replicate measurements were performed. Each measurement consisted of six consecutive runs, each with a duration of 20 s. For every measurement, the relative particle size distribution, intensity-weighted effective diameter (d_eff), and polydispersity index (PDI) were determined. Autocorrelation functions of the scattered intensity were analyzed as described previously (Putaux et al., 2005). For comparative analysis, correlation functions C(q,t) were normalized to their initial value, C(q,0).

### 4.4 Scanning electron microscopy of *Mycoplasma gallisepticum* cells

Cover glasses were plasma-cleaned using a Zepto Plasma Cleaner (Diener, Germany) under a nitrogen atmosphere for 10 min. The surfaces were then treated with a 5% (3-aminopropyl)triethoxysilane (APTES; Macklin, Shanghai, China) solution for 10 min at room temperature (RT). Following silanization, the glasses were rinsed with Milli-Q water (mQ water), and excess liquid was carefully removed using a laboratory wipe.

The treated glasses were subsequently baked at 120 °C for 10 min. After cooling to RT, the surfaces were incubated with a 0.1% glutaraldehyde solution (PanReac AppliChem, Spain) for 10 min. The glasses were then rinsed again with mQ water, and excess liquid was removed.

A 100 μL aliquot of mycoplasma cell suspension was applied to the functionalized glass surface and incubated for 15 min at RT to allow cell adhesion. Cells were then fixed with 2.5% glutaraldehyde for 1 h. Following fixation, the buffer was gradually replaced with a graded ethanol series (35, 50, 70, 80, 96, and 100%), after which ethanol was substituted with acetone. Samples were dried using a critical point dryer (HCP-2, Hitachi, Japan) (Bray, 2000) and sputter-coated with gold.

Imaging was performed using a Quattro S field-emission scanning electron microscope (Thermo Fisher Scientific, United States) operated at an accelerating voltage of 15 kV in secondary electron mode.

### 4.5 Electron paramagnetic resonance spectroscopy

Incorporation of the 16-DSA probe into the *M. gallisepticum* membrane was achieved by incubating cells in cultivation medium lacking horse serum, supplemented with the probe, at 37 °C for 2 h under constant agitation. The probe was added at a ratio of 5 μg per 10^10^ cells. Spin traps (DMPO or PBN) were added immediately prior to analysis at a ratio of 1 μg per 10^10^ cells.

X-band EPR spectra were recorded using a Bruker EMX spectrometer (Bruker, Germany) at the Center for Collective Use “New Materials and Technologies” of the IBCP RAS. The spectrometer was equipped with a variable-temperature control unit. Samples were loaded into glass capillaries with an inner diameter of 1 mm. The final concentration of spin traps in the samples was 100 mM. Spectra were acquired using the following parameters: microwave power, 2 mW; sweep width, 100 G; conversion time, 81.9 ms; time constant, 40.96 ms; modulation amplitude, 0.9 G; and 1,024 data points.

Spectral parameters were determined using WINEPR and SIMPHONIA software packages (Bruker, Germany), as well as the WinSIM program (NIEHS/NIH, United States) (WinSIM (NIEHS/NIH, USA) Program, n.d.). Experimental spectra obtained with the 16-DSA spin probe were analyzed using the “Microscopic Order and Macroscopic Disorder” (MOMD) model implemented in the NLSL program (Budil et al., 1996). This model assumes the presence of locally ordered domains with isotropically distributed directors. Principal values of the *g*-tensor and hyperfine interaction tensor for 16-DSA were adopted from previously published data (Wasserman et al., 2008). During spectral simulation, the hyperfine interaction tensor was varied within a range of 0.5 G to achieve optimal agreement between experimental and theoretical spectra.

### 4.6 Isolation of vesicles from *Mycoplasma gallisepticum* cells

Extracellular vesicles were isolated from *M. gallisepticum* cells as described previously (Wagner et al., 2023). Briefly, mycoplasma cells were removed by centrifugation at 8,000 × g for 30 min. The resulting supernatant was sequentially filtered through 0.45 μm and 0.22 μm pore-size filters (PES membranes; Membrane Solutions, United States). The clarified supernatant was then subjected to ultracentrifugation at 100,000 × g for 2 h at 4 °C using a 45 Ti rotor.

The vesicle-containing pellet was washed with phosphate-buffered saline (PBS) and ultracentrifuged again at 100,000 × g for 2 h at 4 °C using an SW 50.1 rotor. After removal of the supernatant, the vesicle pellet was resuspended in 100 μL of PBS and stored at −80 °C until further analysis.

### 4.7 Determination of hydrogen peroxide production

Hydrogen peroxide production by vesicles derived from *M. gallisepticum* was quantified using the Amplex® Red Hydrogen Peroxide/Peroxidase Assay Kit (Thermo Fisher Scientific Inc., United States) according to the manufacturer’s instructions. Measurements were performed in Krebs–Ringer phosphate buffer.

### 4.8 Peptidase activity assay

Peptidase activity was assessed by incubating 1–2 μg of vesicles with 20 μM of the fluorogenic substrates Suc-LLVY-AMC or Boc-LRR-AMC in a final volume of 20 μL. Fluorescence was measured at an excitation wavelength of 380 nm and an emission wavelength of 445 nm using a microplate reader (Tecan Infinite 200 PRO) at 37 °C. Phosphate-buffered saline (PBS) was used as the assay buffer.

### 4.9 Measurement of ATP concentration in *Mycoplasma gallisepticum* cells and vesicles

ATP was extracted using a methanol-based metabolite extraction protocol as described previously (Vanyushkina et al., 2014). Dried extracts were resuspended in a buffer containing 50 mM HEPES and 160 mM KCl. ATP concentration was quantified using a luciferase-based assay.

The reaction mixture consisted of 100 mM Tris–acetate buffer (pH 7.8), 5 mM MgCl₂, 0.15 mM luciferin (Macklin), ATP (samples or standards), and luciferase (Sigma-Aldrich) at a final concentration of 0.04 mg mL^−1^. The volumetric ratio of reaction mixture, ATP sample, and luciferase in each well was 40:5:5 μL. Chemiluminescence (λ = 560 nm) was measured using a Tecan Infinite 200 PRO microplate reader in white 384-well plates.

Calibration curves were generated using ATP standards ranging from 0.25 to 25 μM. ATP-containing samples were prepared in 50 mM HEPES-K buffer supplemented with 160 mM KCl (pH 7.4). Intracellular ATP content was calculated by normalizing measured ATP levels to the number of *M. gallisepticum* genome copies per sample, as determined by RT-qPCR, and to the mean cell volume (0.3 fL under control conditions and 0.08 fL under stress conditions).

### 4.10 Atomic force microscopy

Freshly cleaved mica sheets (0.5 × 0.5 cm) were treated with 5 mM MgCl₂ for 5 min, thoroughly rinsed with Milli-Q water, and dried under a stream of N₂ gas. Following surface functionalization, vesicle suspensions were diluted 40-fold and deposited onto the mica surface. After incubation for 30 min, samples were gently rinsed with Milli-Q water and dried.

Atomic force microscopy (AFM) measurements were performed at room temperature (22 °C) using an NTEGRA Prima atomic force microscope (NT-MDT, Moscow, Russia). Imaging was carried out in tapping mode using silicon cantilevers (NS15 series; nominal spring constant, 40 N m^−1^) at a scan rate of 1 Hz. The setpoint was optimized individually for each sample to minimize tip-induced damage. AFM data were processed using Image Analysis 1.2.0.0 (Nova SPM 1.2) and Gwyddion 2.64 software.

### 4.11 Overexpression and cell harvesting

Genes encoding *M. gallisepticum* glycolytic enzymes were cloned into the pET-15b(+) expression vector (Invitrogen), generating constructs encoding full-length proteins fused to an N-terminal hexahistidine (6 × His) tag followed by a thrombin cleavage site. Recombinant plasmids were transformed into *Escherichia coli* BL21(DE3) Gold (Invitrogen) competent cells. Transformants were selected on Luria–Bertani (LB) agar plates supplemented with 100 μg mL^−1^ ampicillin. Single colonies were used to inoculate starter cultures, which were aliquoted and stored at −80 °C in LB medium containing 20% (w/v) glycerol.

For large-scale protein expression, LB medium was inoculated with a 1:100 dilution of an overnight pre-culture. Cultures were grown at 37 °C with shaking at 180 rpm until an optical density at 600 nm (OD₆₀₀) of approximately 0.6 was reached. Protein expression was induced by addition of isopropyl β-D-1-thiogalactopyranoside (IPTG) at final concentrations specified for each enzyme in Supplementary Table S3 (range: 0.1–1 mM). Induction temperature and duration for each enzyme are also detailed in Supplementary Table S3.

Cells were harvested by centrifugation at 4,000 × g for 20 min at 4 °C. The resulting pellets were resuspended in ice-cold wash buffer (50 mM Tris–HCl, pH 7.5, 200 mM NaCl) and centrifuged again under the same conditions. Cell pellets were subsequently stored at −20 °C until further use.

### 4.12 Cell lysis and clarification

For cell lysis, frozen cell pellets were thawed on ice and resuspended in ice-cold lysis buffer at a ratio of 10 mL per gram of wet cell weight. The lysis buffer consisted of 20 mM Tris–HCl, 1,000 mM NaCl, 5% (v/v) glycerol, 2 mM phenylmethylsulfonyl fluoride (PMSF), and 1 mM dithiothreitol (DTT), with pH values specified in Supplementary Table S3. Lysozyme was added to a final concentration of 0.1 mg mL^−1^, and suspensions were incubated on ice for 15 min. Cells were then disrupted by ultrasonication using a Scientz-IIID homogenizer at 80% amplitude. Sonication was performed in 2–3 cycles of 7 min each, consisting of 5 s pulses followed by 7 s pauses, with 5 min cooling intervals on ice between cycles. Cell lysates were clarified by centrifugation at 30,000 × g for 30 min at 4 °C to remove cellular debris, unlysed cells, and membrane fragments. The resulting supernatant was filtered through a 0.22 μm membrane.

The clarified lysate was loaded onto a Ni–NTA affinity column (5 mL bed volume; Sepax Technologies) pre-equilibrated with lysis buffer. The column was washed sequentially with 30 column volumes (CV) of lysis buffer followed by 30 CV of wash buffer containing 50 mM Tris–HCl, 200 mM NaCl, 20 mM imidazole, 5% (v/v) glycerol, 2 mM PMSF, and 1 mM DTT, with pH values specified in Supplementary Table S3. Bound proteins were eluted using elution buffer identical to the wash buffer but supplemented with 500 mM imidazole. Protein-containing fractions were pooled and concentrated using Amicon Ultra-4 centrifugal filters with a 10 kDa molecular weight cutoff (Millipore).

Concentrated protein samples were centrifuged at 20,000 × g for 10 min at 4 °C to remove aggregates and subsequently applied to a Bio-Gel P-6 desalting column (Bio-Rad) pre-equilibrated with storage buffer (Supplementary Table S3) and connected to a Bio-Lab 30 chromatography system (Hanbon). Peak fractions containing protein were pooled and further concentrated using Amicon Ultra-4 centrifugal filters (10 kDa MWCO). Protein concentration was determined by the Bradford assay using bovine serum albumin as a standard. Purified proteins were supplemented with glycerol to a final concentration of 50% (v/v), aliquoted, and stored at −20 °C.

### 4.13 *In vitro* glycolysis kinetic measurement

All assays were performed in buffer containing 50 mM HEPES-K, 160 mM KCl, and 50 mM MgCl₂ at 25 °C. In the *in vitro* reconstruction of glycolysis, the phosphotransferase system (PTS) sugar transporter was not included because it is a membrane protein. Reactions were initiated by addition of glucose-6-phosphate to reaction mixtures containing 5 mM NAD^+^, 1 mM ADP, 1 mM dithiothreitol (DTT), and 10 mM potassium phosphate buffer (KH₂PO₄/K₂HPO₄, pH 7.4). ATP concentrations were varied between 5 mM, 15 mM, and 30 mM.

Glycolytic kinetics were monitored by measuring the increase in NADH absorbance at 340 nm using a Tecan Infinite 200 PRO microplate reader. A calibration curve was generated using NADH standards ranging from 10 μM to 1 mM prepared in reaction buffer. Continuous measurements were recorded for 140 min.

Enzyme stoichiometries used for *in vitro* reconstitution of the glycolytic pathway were selected based on quantitative proteomic profiles of *M. gallisepticum* cells under control, heat shock, and hypoosmotic conditions (Figure 5A; Supplementary Table S4).

Kinetic parameters were determined under steady-state conditions. Initial reaction rates (v₀) were calculated from the linear portion of each kinetic curve corresponding to less than 5% substrate conversion. Michaelis–Menten parameters were obtained by nonlinear regression of v₀ as a function of substrate concentration [S] using the Equation 3:


$$\;\;v_{0}=\frac{V_{\max}[S]}{K_{m}+[S]}\;\;\;\;$$ (3)

where *V*_max_ is the maximal reaction velocity and *K*_m_ is the Michaelis constant (Lorsch, 2014; Srinivasan, 2022).

Substrate inhibition kinetics were described using the following model (Wang et al., 1999) (Equation 4):


$$\;v=\frac{V_{\max}[S]}{K_{m}+[S]+\frac{{\left[S\right]}^{2}}{K_{i}}}\;\;$$ (4)

where [*S*] is the substrate concentration, *V*_max_ is the maximal velocity, *K*_m_ is the Michaelis constant, and *K*ᵢ is the inhibition constant.

### 4.14 Enzyme encapsulation into liposomes

Liposomes were prepared using the thin film hydration method with slight modifications. Briefly, lipids were aliquoted from chloroform stock solutions into a vessel and spread evenly to form a thin layer along the walls. The solvent was evaporated under a stream of dry argon for 1 h, followed by further drying under reduced pressure (5 mbar) for 1 h at room temperature. The resulting dry lipid film was hydrated by exposure to a gentle stream of argon that had been bubbled through 300 mL of distilled water heated to 50 °C for 1 h. The vessel containing the pre-hydrated film was then frozen in liquid nitrogen, and 500 μL of internal buffer (containing enzymes and substrates) was added, yielding a final lipid composition of 4 g/L 1-palmitoyl-2-oleoyl-glycero-3-phosphocholine (POPC), 1 g/L 1,2-di-(9Z-octadecenoyl)-sn-glycero-3-phospho-L-serine (sodium salt) (DOPS), and 0.005 g/L 1,2-dioleoyl-sn-glycero-3-phosphoethanolamine-N-(lissamine rhodamine B sulfonyl) (ammonium salt) (Liss Rhod PE). All components from Avanti Polar Lipids (Alabaster, United States). After brief vortexing until the suspension appeared homogeneous, it was subjected to 10 freeze–thaw cycles (freezing in liquid nitrogen and thawing in a room-temperature water bath). The resulting suspension was extruded 21 times through polycarbonate membranes with 1 μm pores (Cytiva, Washington, United States) using an Avanti Mini Extruder (Alabaster, United States). The obtained liposomes (300 μL) were immediately transferred to a pre-cooled (4 °C) size exclusion chromatography column packed with pre-equilibrated Smartdex G-75 resin (Smart-Lifesciences, Changzhou, PRC). After elution, the liposomal fraction was identified based on rhodamine fluorescence and collected to obtain a final liposomal dispersion free of unencapsulated enzymes.

To assess the uniformity of protein encapsulation and its spatial distribution within the liposomes, we performed laser confocal scanning microscopy (LCSM). Following an identical protocol to that used for the enzymes, we encapsulated the green fluorescent protein (GFP). Subsequent LCSM imaging revealed the distribution of GFP fluorescence throughout the interior aqueous volume of liposomes, confirming successful and uniform protein loading.

The LCSM images show a population of small particles with some larger aggregates exceeding 1 μm (Supplementary Figure S8A). Although unloaded liposomes, which register only in the red channel (Liss Rhod PE), can be distinguished, the majority of the vesicles exhibit noticeable green emission, confirming substantial GFP encapsulation. A closer inspection of the images allowed us to estimate the diameter of the majority of the liposomes to be in the range of 400–900 nm (Supplementary Figure S8B).

Hydrodynamic diameter of N-Lip liposomes was evaluated by DLS on a 90plus particle analyzer (Brookhaven Instruments, Nashua, United States) in disposable plastic cuvettes. Average diameter was 398 ± 40 nm with polydispersity index of 0.23 ± 0.10.

### 4.15 Kinetic characterization of glycolysis in liposomes

A minimal glycolytic system was reconstituted inside liposomes to study metabolism in a confined environment. The internal liposome solution, composed of a modified Mycoplasma-like buffer (20 mM HEPES-Na, 15.5 mM K_2_HPO_4_, 4.5 mM KH₂PO₄, 160 mM KCl, 2 mM MgCl₂, 10 mM sucrose; pH 7.4), contained a stoichiometric mixture (0.25 μM each) of recombinant *M. gallisepticum* glycolytic enzymes (from GPI to PYK) and supplemented with exogenous *E. coli* hexokinase to initiate the pathway. The system was provided with essential substrates: 5 mM ATP, 1 mM ADP, 5 mM NAD+, 1 mM DTT, and 2 mM glucose. Liposomes were formed using thin film hydration method detailed in the previous section and were suspended in an isoosmotic external buffer, which was identical to the internal buffer except for a higher KCl concentration (165 mM) to balance osmolality.

The kinetic analysis of glycolytic activity was performed by monitoring NADH production in freshly prepared liposome suspensions (1 mL). Fluorescence measurements were carried out using a Hitachi F-2500 fluorometer (Tokyo, Japan) equipped with 1 × 1 cm quartz cuvettes. The excitation wavelength was set to λ_ex_ = 365 nm, λ_em_ = 450 nm with a 10 nm slit width.

The resulting kinetic curves of NADH fluorescence were fitted to a first-order exponential growth model to determine the characteristic reaction time (*τ*) via the following Equation 5:


$$I_{\text{fluo}}=I_{\text{fluo}}^{0}+A\cdot (1-\exp(-\frac{t-\mathrm{TD}}{\tau }))$$ (5)

where *I_fluo_* is the fluorescence intensity at time *t*, *I*^0^*_fluo_* is the initial fluorescence intensity; *A* is the amplitude (maximum fluorescence change); *TD* is the time delay; *τ* is the characteristic time constant; *t* is the reaction time.

### 4.16 Assessment of liposome compression under hyperosmotic stress by calcein fluorescence quenching

To assess the physical response of liposomes to osmotic stress, we employed a calcein fluorescence quenching assay. Calcein was loaded into the liposomes using the same encapsulation procedure as for the enzymes. Fluorescence was then measured under isosmotic and hyperosmotic conditions. A decrease in fluorescence intensity under hyperosmotic conditions indicates liposome compression and self-quenching of the encapsulated calcein.

The magnitude of fluorescence intensity decrease can be used to evaluate unknown calcein concentration by linear interpolation of the self-quenching calibration curve (Supplementary Figure S9). The obtained concentration change, which was 178 μM (initial calcein concentration was 100 μM), allows to evaluate volume in hyperosmotic conditions as 56% of the initial value. This is expected, since the osmolality of the outer solution is exactly twice higher than for isosmotic conditions.

### 4.17 Proteomic analysis

Samples were incubated with ProteinSafe™ Protease Inhibitor Cocktail (TransGen, China) and benzonase (diaGene, Russia; 5 U per 100 μL of sample) for 10 min at 37 °C, followed by incubation with 4% SDS for 30 min at 60 °C. Samples were then lysed by sonication at 40% amplitude using a pulse regime of 15 s on and 30 s off for a total processing time of 3 min, with the temperature maintained at 4 °C. Lysates were clarified by centrifugation at 15,000 × g for 10 min, and the supernatants were transferred to fresh tubes.

Proteins were precipitated using a chloroform–methanol extraction protocol. Briefly, 400 μL of methanol was added to 100 μL of protein sample in a 1.5 mL microcentrifuge tube and mixed thoroughly by vortexing. Subsequently, 100 μL of chloroform was added, followed by additional vortexing. Next, 300 μL of Milli-Q water was added, and the mixture was vortexed and centrifuged at 14,000 × g for 1 min. The upper aqueous phase was carefully removed. Proteins were then precipitated by addition of 400 μL of methanol, followed by vortexing and centrifugation at 20,000 × g for 5 min. The supernatant was discarded, and the resulting protein pellet was dried under vacuum.

Precipitated proteins were denatured for 30 min in buffer containing 8 M urea, 2 M thiourea, and 10 mM Tris (pH 8.0). Protein concentration was determined using a Bradford assay, and 200 μg of protein from each sample was used for subsequent analysis. Disulfide bonds were reduced with 15 mM dithiothreitol (DTT) at 56 °C for 30 min and alkylated with 30 mM iodoacetamide for 30 min at room temperature in the dark. The reduction step with DTT was then repeated, after which the reaction mixture was diluted 6-fold with 50 mM Tris–HCl (pH 8.0).

Proteolytic digestion was performed using Trypsin Gold (mass spectrometry grade; Promega) at an enzyme-to-protein ratio of 1:50 (w/w) overnight at 37 °C. Digestion was terminated by acidification with trifluoroacetic acid (TFA) to a final concentration of approximately 1%. The resulting peptide mixture was desalted using Copure C18A solid-phase extraction (SPE) cartridges (100 mg mL^−1^; Biocomma), dried under vacuum, and reconstituted in 5% acetonitrile (ACN) containing 0.1% formic acid (FA) prior to LC–MS/MS analysis.

LC–MS/MS analysis of peptide samples was performed using an Orbitrap Exploris 480 mass spectrometer (Thermo Scientific, United States) coupled to an Ultimate 3000 RSLCnano chromatographic system (Dionex, United States) via a Nanospray Flex ion source (Thermo Scientific, United States).

Peptide samples were resuspended in 35 μL of 5% acetonitrile (HPLC grade) in water (LC–MS grade) containing 0.1% (v/v) formic acid. Chromatographic separation was performed by reversed-phase liquid chromatography using a C18 PepMap 100 trap column (C18 sorbent, 5 mm length, 300 μm inner diameter, 5 μm particle size, 100 Å pore size; Thermo Scientific, United States) coupled to a Peaky-C18 analytical capillary column (C18 sorbent, 50 cm length, 75 μm inner diameter, 1.9 μm particle size; Molekta, Russia).

For each analysis, 5 μL of sample was loaded onto the trap column using 5% acetonitrile (HPLC grade) in water (LC–MS grade) with 0.1% (v/v) formic acid at a flow rate of 10 μL min^−1^ for 12 min. The trap column was then switched in line with the analytical column. Peptides were eluted using a binary solvent system consisting of solvent A (5% acetonitrile in water, 0.1% formic acid, v/v) and solvent B (79.9% acetonitrile, 20% water, 0.1% formic acid, v/v).

The gradient was applied at a flow rate of 250 nL min^−1^ as follows: solvent B was increased from 5 to 10% over 3 min, from 10 to 55% over 54 min, and from 55 to 65% over 3 min. After peptide elution, the system was washed with 100% solvent B for 2 min and subsequently re-equilibrated with 5% solvent B for 15 min.

The electrospray ionization source was operated at a spray voltage of 2,700 V, and the capillary temperature was set to 275 °C. The mass spectrometer was operated in data-dependent acquisition (DDA) mode. In each acquisition cycle of 1 s, a full MS survey scan was recorded, followed by automatic selection of precursor ions for fragmentation and acquisition of MS/MS spectra. Precursor ion selection was performed automatically, and dynamic exclusion was enabled to prevent repeated fragmentation of ions for which MS/MS spectra had already been acquired.

Full MS spectra were acquired at a resolution of 60,000 over an *m/z* range of 200–1,500, with the automatic gain control (AGC) setting set to “Standard” and the maximum injection time set to “Auto.” The Funnel RF level was set to 50. MS/MS spectra were acquired at a resolution of 15,000 with the AGC setting “Standard” and an automatic injection time. Fragmentation was performed using a normalized collision energy of 30, with an isolation window of 1.4 *m/z*.

Quantitative analysis of MS/MS data was performed using MaxQuant software (Cox and Mann, 2008), with peptide identification carried out by the Andromeda search engine against the *Mycoplasma gallisepticum* S6 genome annotation database. For each sample, relative protein abundance was calculated as the ratio of the protein’s intensity-based absolute quantification (iBAQ) value to the summed iBAQ intensity of all detected mycoplasma proteins in that sample. Within each experimental group, median relative intensities across biological replicates were calculated, and fold changes were determined relative to the control condition. Statistical significance was assessed using *p* values adjusted for multiple testing as described previously (Benjamini and Hochberg, 1995).

### 4.18 Lipid extraction and MS analysis

Lipids from 50 mL cultures of *M. gallisepticum* were extracted using the Bligh and Dyer method (Bligh and Dyer, 1959). Dried lipid extracts were thoroughly resuspended in 10 μL of 7.5 mM ammonium acetate prepared in chloroform/methanol/propanol (1:2:4, v/v/v). The volume of each resuspended sample was normalized to protein content and adjusted to a final volume of 500 μL using the same solvent mixture prior to direct infusion into an Orbitrap Exploris 480 mass spectrometer (Thermo Scientific, United States) equipped with an OptaMax NG ion source (Thermo Scientific, United States). Samples were infused at a flow rate of 5 μL min^−1^.

Analyses were performed in positive ion mode at the MS^1^ level only, with a resolving power of 60,000. The spray voltage was set to 3,400 V in positive ion mode, and the capillary temperature was set to 320 °C. In the absence of chromatographic separation, sensitivity was increased by acquiring data in consecutive *m/z* windows of 50 m/z width (300–350, 350–400, 400–450, 450–500, 500–550, 550–600, 600–650, continuing up to 1,150–1,200 m/z). The acquisition window was switched every 10 s, and the microscans parameter was set to 10.

Raw data files were converted to mzML format using MSConvert (version 3.0.25239-6532e25). Lipid identification and quantification were performed using LipidXplorer (version 1.2.9) with a mass tolerance of 2 ppm, an absolute intensity threshold of 2,500, and a resolution gradient of −36.11 res/(m/z). A total of 22 MFQL query files were used for lipid identification.

All analyses were performed using three biological replicates. Normalization between samples was performed by measuring *M. gallisepticum* genome copies per sample, as determined by RT-qPCR. Statistical significance was assessed using *p* values adjusted for multiple testing as described previously (Benjamini and Hochberg, 1995).

### 4.19 VlhA *in silico* analyses

VlhA protein structures were predicted using AlphaFold^2^ (Jumper et al., 2021) and visualized with PyMOL (version 2.5.4).

### 4.20 Statistical analysis

Hydrogen peroxide production, ATP levels, peptidase activity, and kinetic characterization of glycolysis in liposomes are presented as mean ± standard deviation (SD) from three technical replicates, and the experiments were performed in three biological replicates. Statistical significance between experimental groups was assessed using Student’s *t* test. Differences were considered statistically significant at *p* < 0.05. For SEM analysis, two biological replicates were examined, each with two technical replicates.

## Data Availability

The datasets presented in this study can be found in online repositories. The names of the repository/repositories and accession number(s) can be found in the article/Supplementary material.
