# Supplementary material for: When less is more: cell size reduction as a metabolic lifeline in Mycoplasma gallisepticum
Source: Front Microbiol. 2026 Jun 30;17:1825835. doi: 10.3389/fmicb.2026.1825835 (PMC13440150; doi:10.3389/fmicb.2026.1825835)
Supplement: Supplementary file 1 [file Data_Sheet_1.pdf]

Supplementary Material

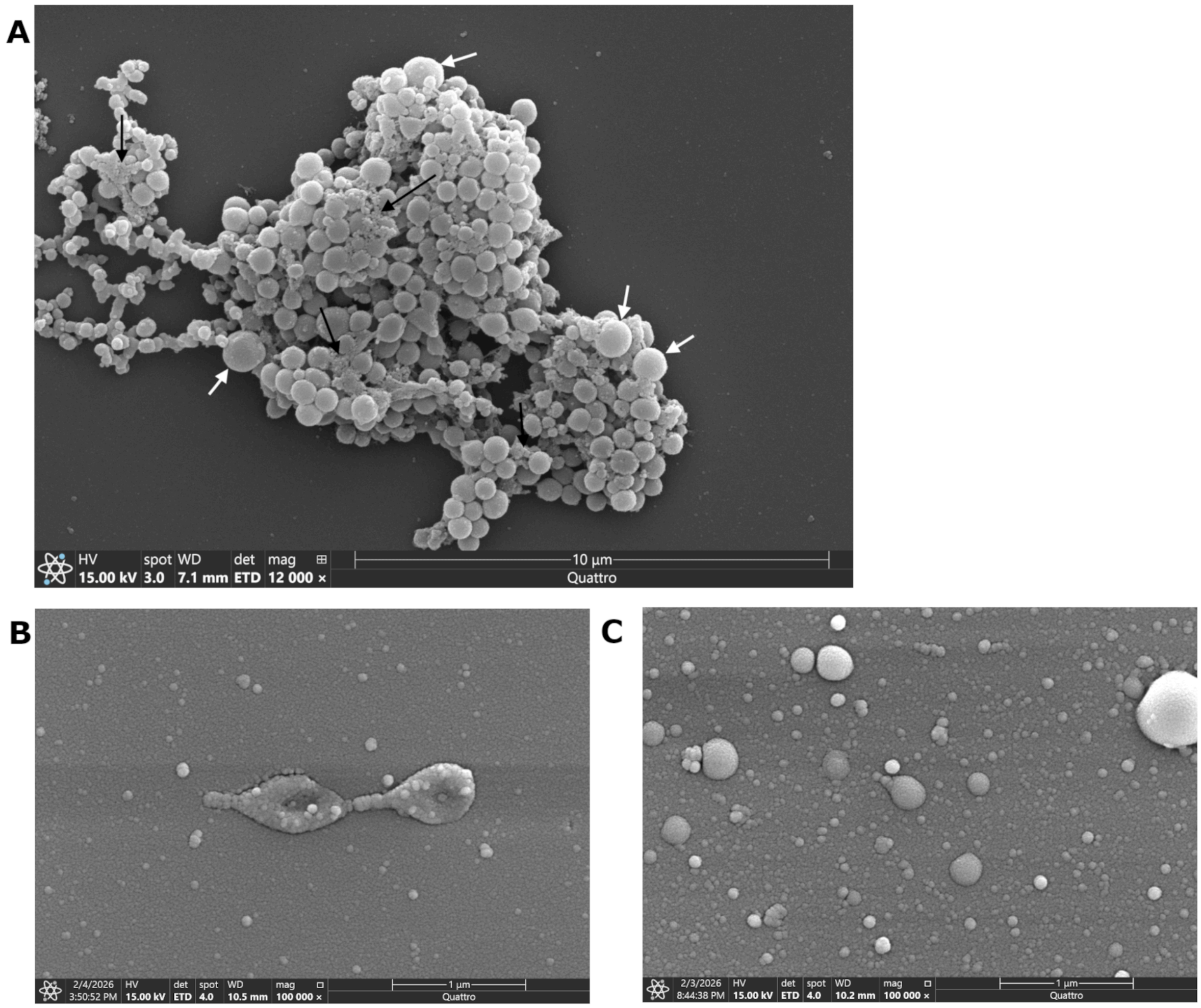

Supplementary Figure 1.

Scanning electron microscopy images of mycoplasma cells. **(A)** *M. gallisepticum* cells and their derived vesicles under hyperosmotic stress. Swollen cells are indicated by white arrows, and vesicles are indicated by black arrows; **(B)** *M. pneumoniae* cells, control conditions; **(C)** *M. pneumoniae* cells, heat shock (42 °C, 30 min).

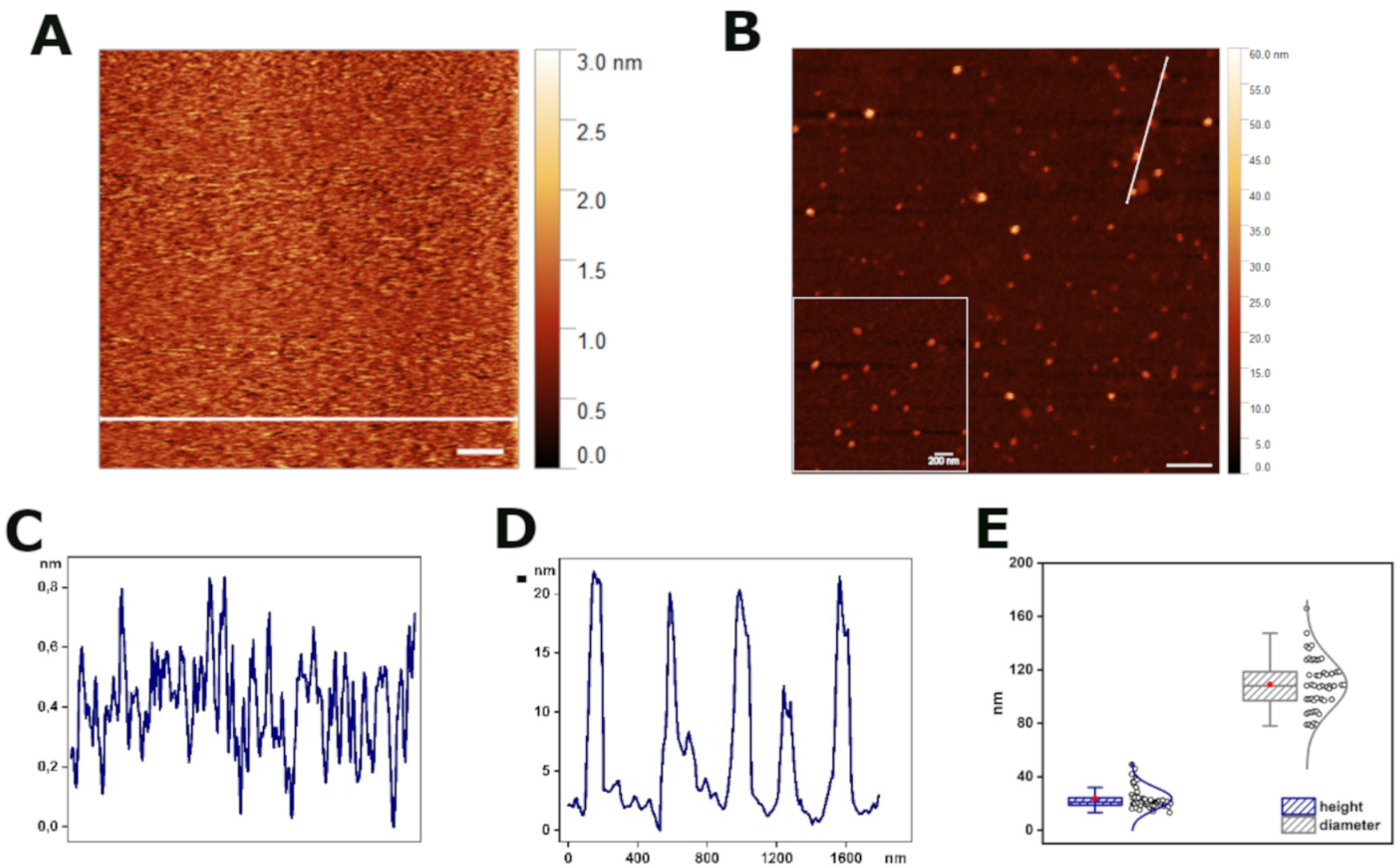

Supplementary Figure 2.

Atomic force microscopy (AFM) analysis of the vesicle-enriched fraction from *M. gallisepticum* cultures. **(A, B)** AFM images of control samples treated with $MgCl_2$ (**A**) and isolated vesicles (**B**). **(C, D)** Corresponding height profiles measured along the white lines indicated in panels **(A)** and **(B)**, respectively. Scale bar, 500 nm. Inset is high magnification image. Particles were deposited onto freshly cleaved mica surfaces modified with 5 mM $MgCl_2$. Vesicles appeared as globular structures, and only particles with heights greater than 10 nm were included in the analysis. **(E)** Distribution of vesicle height and diameter values for *M. gallisepticum* vesicles deposited on modified mica, as determined by AFM. Mean vesicle diameter was 108.97 ± 19.33 nm, and mean height was 23.69 ± 8.15 nm. In the box plot, the red dot indicates the mean value, the horizontal line within the box represents the median, and the upper and lower whiskers correspond to the 99th and 1st percentiles, respectively.

A

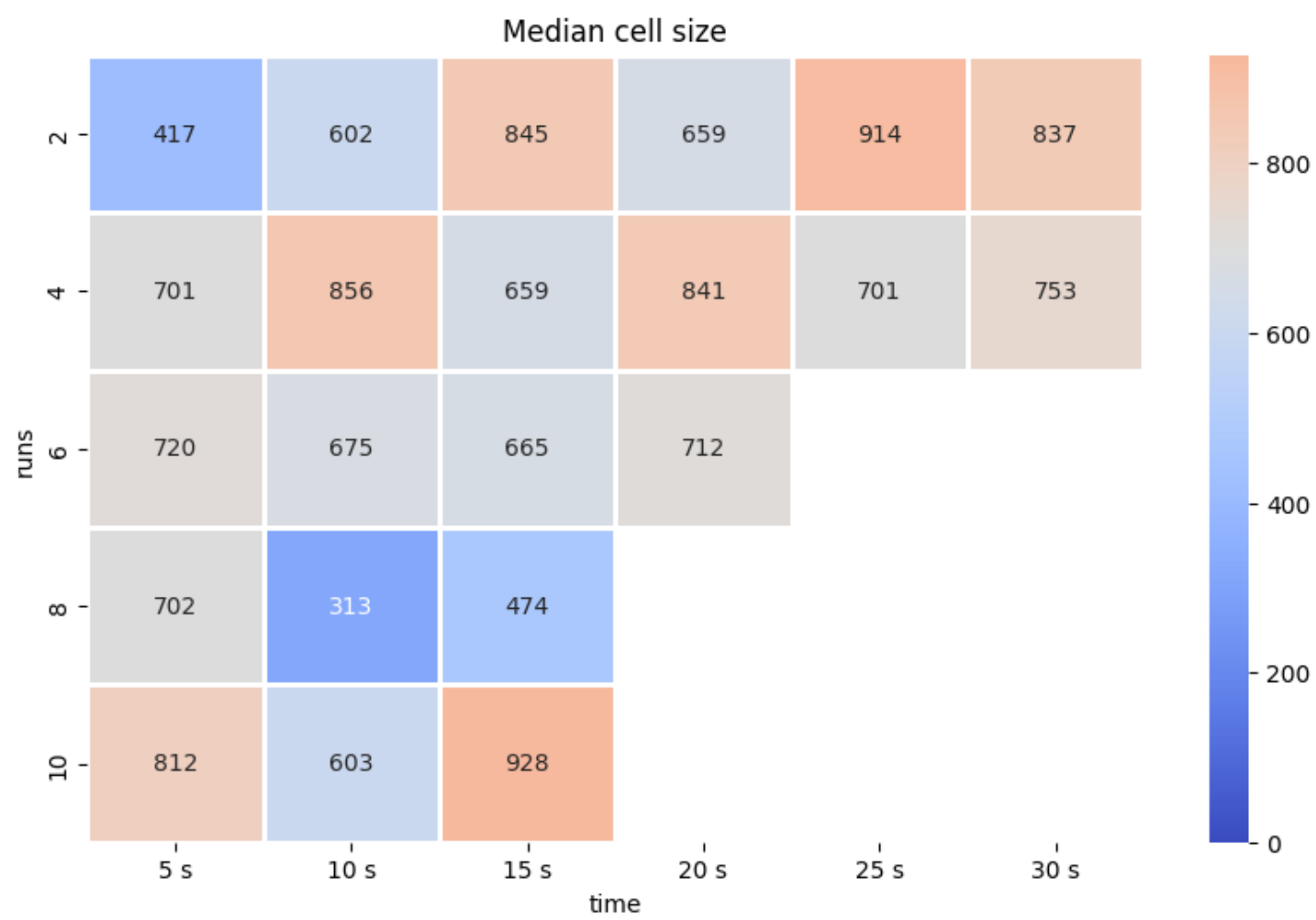

B

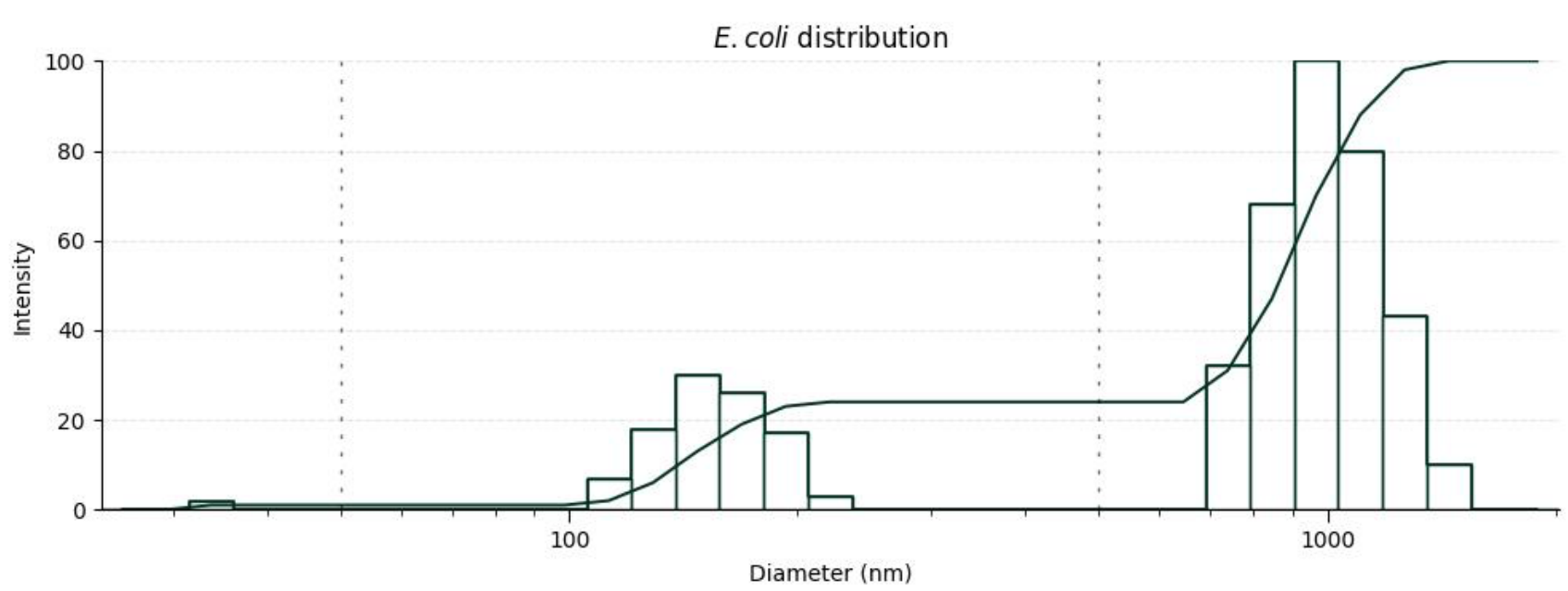

C

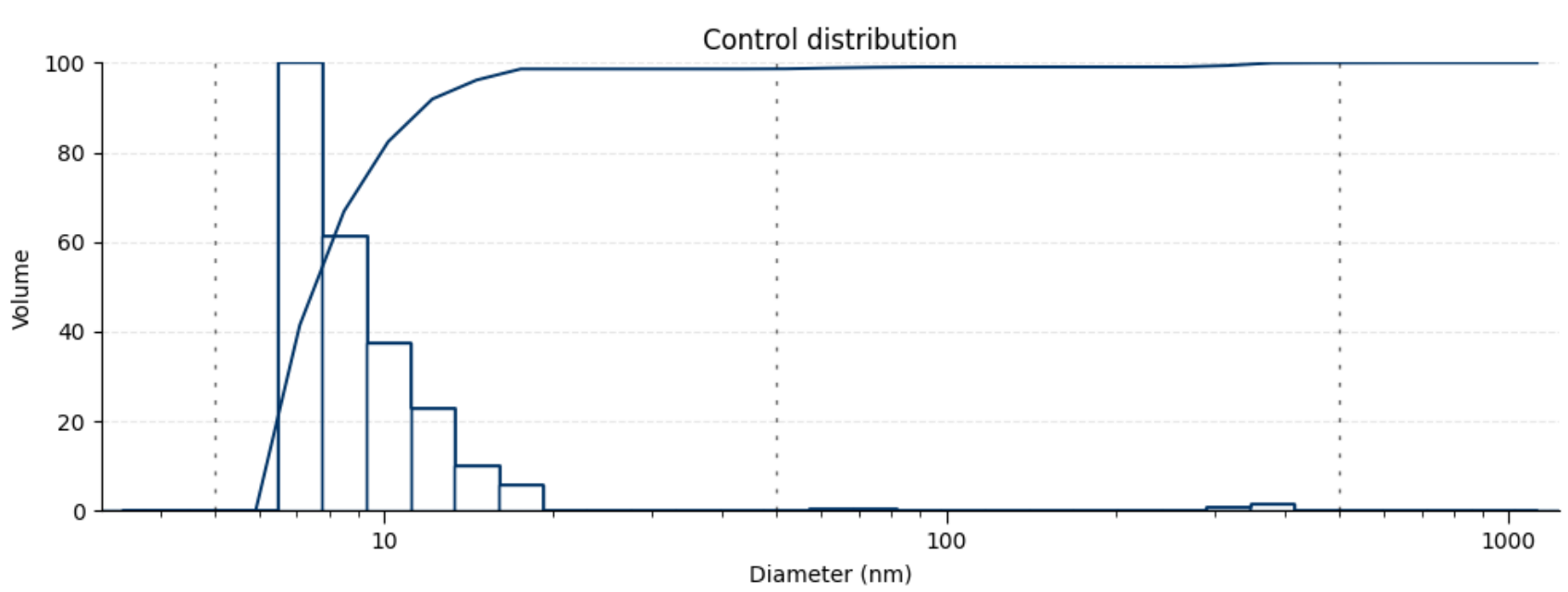

Supplementary Figure 3.

Control experiments for bacterial cell sizing by dynamic light scattering (DLS). **(A)** Matrix of linear size measurements for control *M. gallisepticum* cells obtained by DLS as a function of the number of acquisition runs and run duration. Stable size estimates corresponding to the true dimensions of *M. gallisepticum* cells were achieved using an acquisition mode consisting of six consecutive runs with a duration of 20 s each; **(B)** Distributions of linear cell sizes for *E. coli* cells; **(C)** Volume-weighted size distributions of control *Mycoplasma gallisepticum* sample.

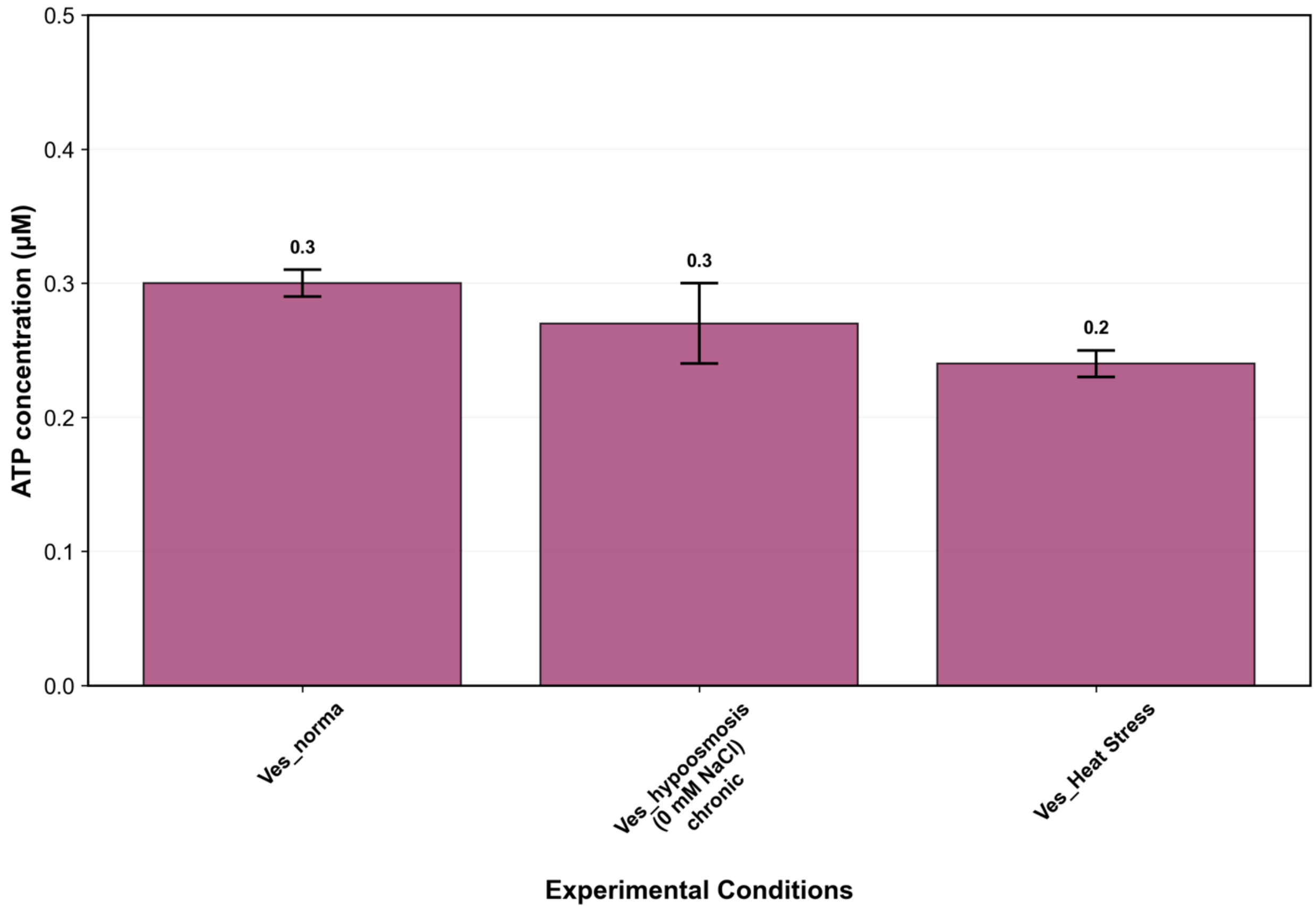

Supplementary Figure 4.

ATP content in vesicle-enriched fractions isolated from *M. gallisepticum* cultures under different stress conditions. ATP levels were measured using a luciferin–luciferase assay and normalized to total protein content. Statistical significance between experimental groups was assessed using Student's t test. Differences were considered statistically significant at $P < 0.05$.

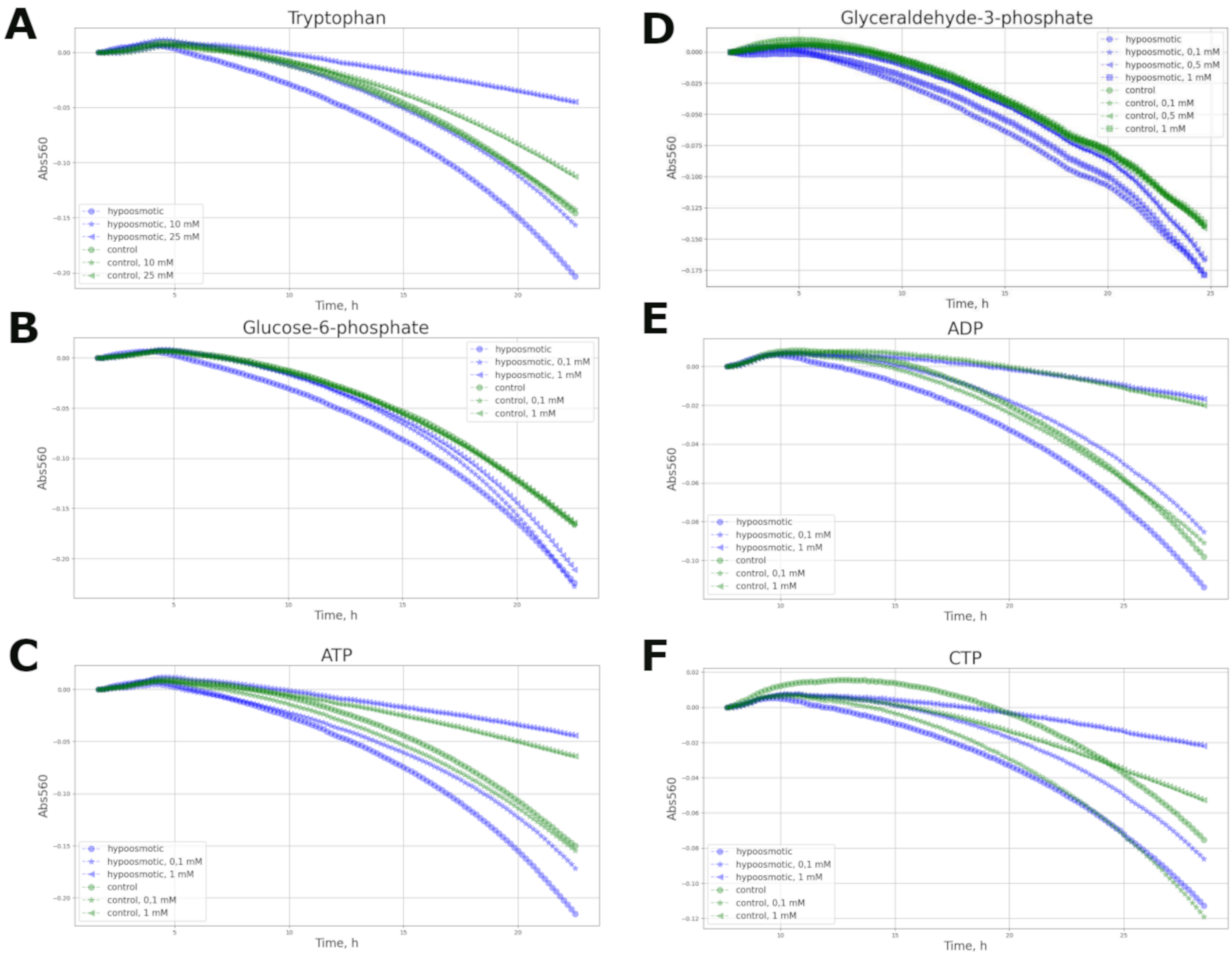

Supplementary Figure 5.

Effects of metabolites on *M. gallisepticum* growth under hypoosmotic and normosmotic conditions. Growth responses to tryptophan (**A**), glucose-6-phosphate (G-6P; **B**), ATP (**C**), glyceraldehyde-3-phosphate (G3P; **D**), ADP (**E**), and CTP (**F**) are shown.

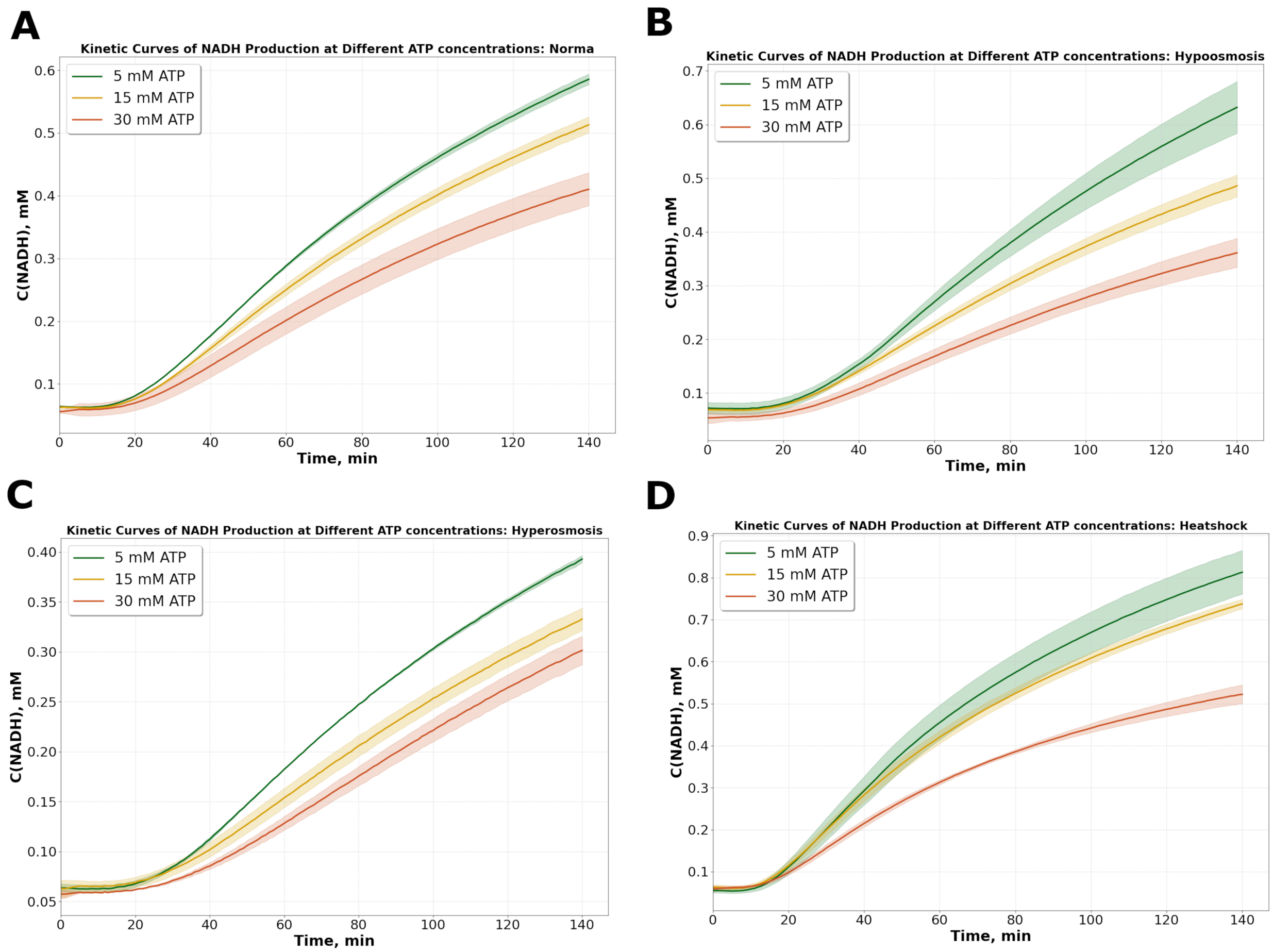

Supplementary Figure 6.

In vitro NADH production kinetics measured over an ATP concentration range of 5–15 mM using glycolytic protein ratios corresponding to control (**A**), hypoosmotic (**B**), hyperosmotic (**C**), and heat shock (**D**) conditions.

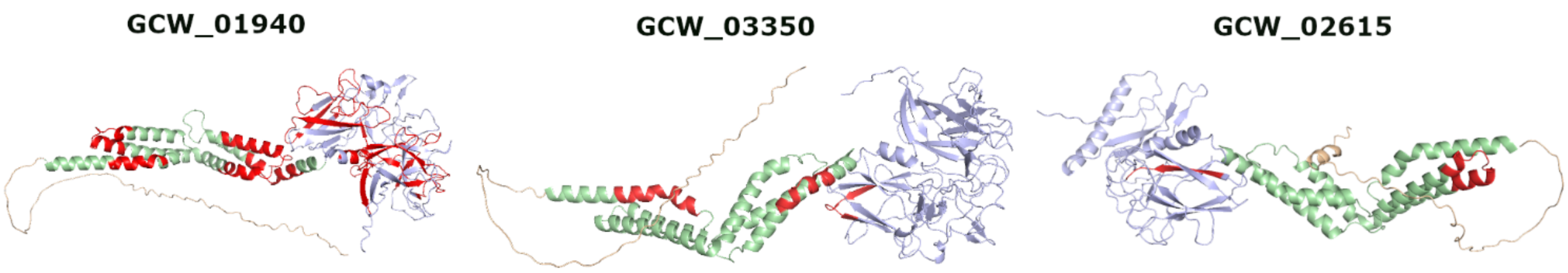

Supplementary Figure 7.

Localization of Vlh antigen peptides identified by mass spectrometry in stress-induced vesicles from *M. gallisepticum*. Peptides identified in this study are highlighted in red. Structural features are color-coded as follows: intracellular N-terminus (yellow), transmembrane α-helices (green), and extracellular C-terminus (purple).

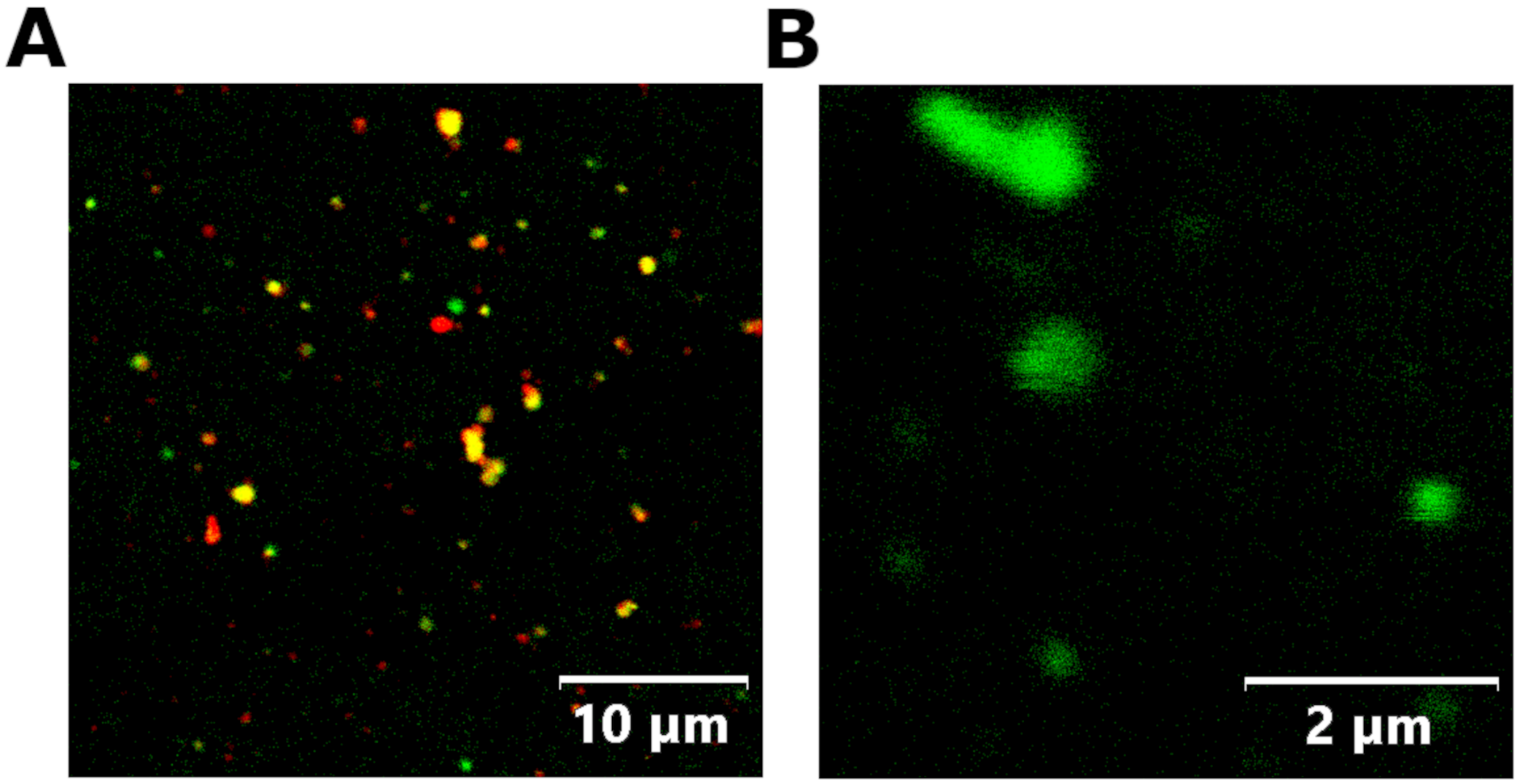

Supplementary Figure 8.
LSCM analysis of N-Lip liposomes. **(A)** General view of the liposome population, showing predominantly small unilamellar vesicles with occasional large aggregates exceeding 1 µm. **(B)** Zoomed-in image revealing individual liposomes with diameters in the range of 400-900 nm.

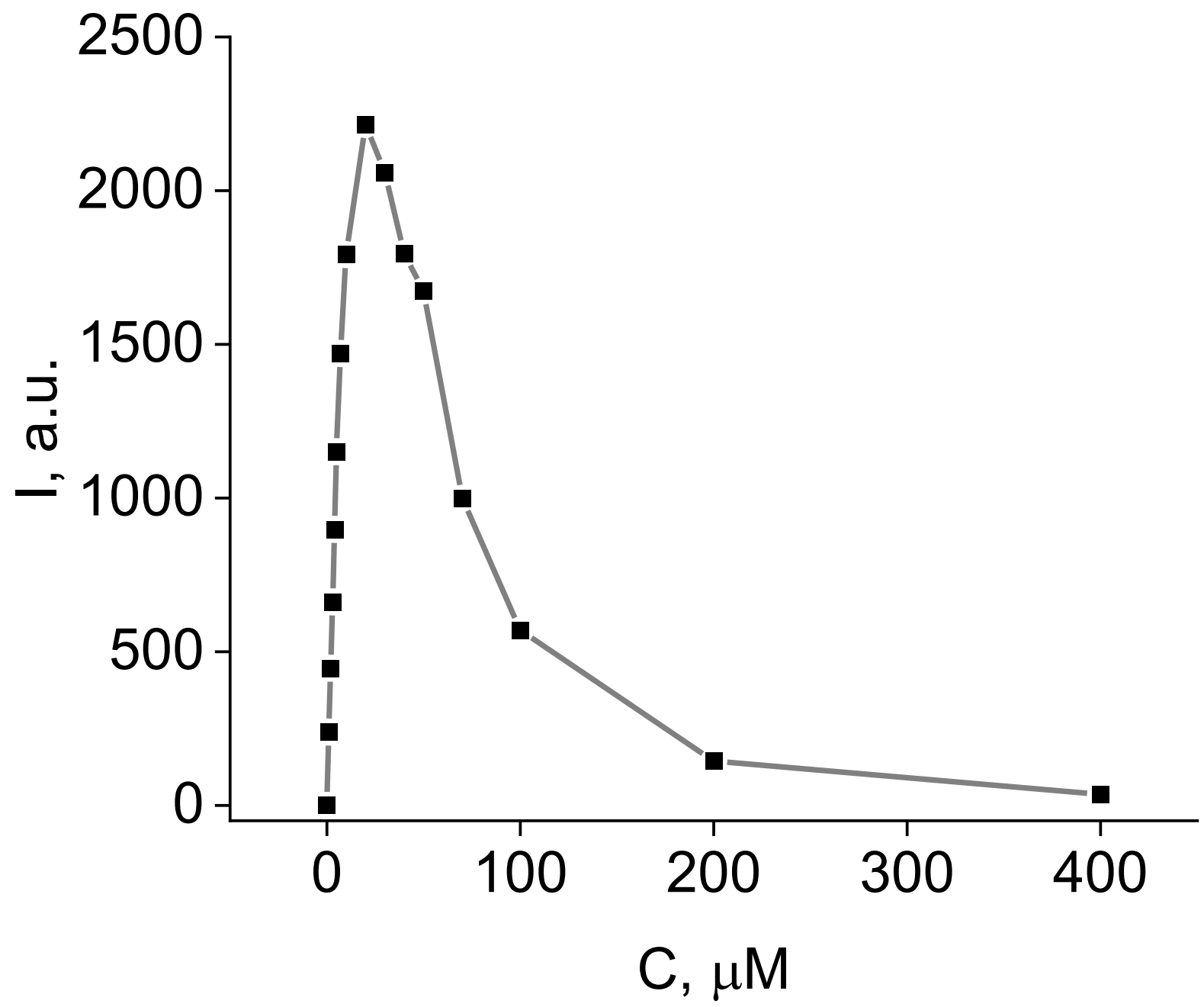

Supplementary Figure 9.

Self-quenching of calcein fluorescence upon concentration increase.

Supplementary Table 3.

Expression parameters for recombinant glycolytic enzymes of *M. gallisepticum.*

Enzyme	IPTG conc. (mM)	Expression time (h)	Expression temp (°C)	Shaking (rpm)	Theor. pI (Expasy)	M.w. (kDa)	Ni-NTA, buffers pH	Storage buffer
**GPI**	0.1	16-20	18	180	8,95	50,9	7	20 mM HEPES, 180 mM NaCl, pH = 7.0, 1 mM DTT, 1 mM PMSF, 50% v/v glycerol
**PFK**	0.1	16-20	18	180	6,9	38,7	8	20 mM HEPES 200 mM NaCl pH = 8.1, 1 mM MgSO4, 1 mM DTT, 1 mM PMSF, 50% v/v glycerol
**FBA**	1.0	16-20	18	180	6,95	33,37	8,35	50 mM TRIS-HCl, 180 mM NaCl, pH = 8.0, 1 mM PMSF, 1 mM DTT, 50% v/v glycerol
**TPI**	1.0	6	24	180	8,37	30,358	8,8	50 mM TRIS-HCl, 180 mM NaCl, pH = 7.0, 1 mM PMSF, 1 mM DTT, 50% v/v glycerol
**GAPD(H)**	0.1	16-20	18	180	9,16	39,37	6,8	20 mM HEPES, 180 mM NaCl, pH = 7.5, 1 mM PMSF, 2 mM DTT, 50% v/v glycerol
**PGK**	0.1	16-20	18	180	6,91	48	8,5	20 mM HEPES, 180 mM NaCl, pH = 8.0, 1 mM DTT, 1 mM PMSF, 50% v/v glycerol
**PGM**	0.1	16-20	18	180	6,68	58,95	8	20 mM HEPES, 180 mM NaCl, pH = 8.0, 1 mM DTT, 1 mM PMSF, 50% v/v glycerol
**ENO**	0.1	16-20	18	180	9,38	53,6	8	20 mM HEPES, 180 mM NaCl, pH = 7.4, 1 mM DTT, 1 mM PMSF, 50% v/v glycerol

**PYK**	0.1	16-20	18	180	7	60,2	8,5	20 mM HEPES, 180 mM NaCl, pH = 8.1, 1 mM DTT, 1 mM PMSF, 50% v/v glycerol
**LDH**	0.1	16-20	18	180	8,25	37,85	7	20 mM HEPES, 180 mM NaCl, pH = 7.0, 1 mM DTT, 1 mM PMSF, 50% v/v glycerol

Supplementary Table 4.

Enzyme stoichiometry (μM in reaction mixture) for *in vitro* glycolysis reconstitution under different stress conditions in *M. gallisepticum* cells.

Protein IDs	Protein names	Heat shock	Hypoosmosis (0 mM NaCl)	Hyperosmosis (250 mM NaCl)	Control
A0A0F6CLB9_MYCGL	Glucose-6-phosphate isomerase (GPI)	0,111	0,082	0,048	0,066
A0A0F6CKX2_MYCGL	6-phosphofructokinase (PFK)	0,142	0,093	0,085	0,081
A0A0F6CLE2_MYCGL	Fructose-bisphosphate aldolase (FBA)	0,164	0,078	0,076	0,068
A0A0F6CL68_MYCGL	Triosephosphate isomerase (TPI)	0,279	0,17	0,191	0,162
A0A0F6CKH9_MYCGL	Glyceraldehyde-3-phosphate dehydrogenase (GAPD)	0,368	0,25	0,147	0,221
A0A0F6CKI0_MYCGL	Phosphoglycerate kinase (PGK)	0,186	0,141	0,134	0,13
A0A0F6CL67_MYCGL	2,3-bisphosphoglycerate-independent phosphoglycerate mutase (PGM)	0,116	0,118	0,123	0,11
A0A0F6CKZ5_MYCGL	Enolase (ENO)	0,146	0,202	0,037	0,178
A0A0F6CKX1_MYCGL	Pyruvate kinase (PYK)	0,106	0,097	0,019	0,075
A0A0F6CJX6_MYCGL	Lactate dehydrogenase (LDH)	0,25	0,25	0,25	0,25
